# How to study runs of homozygosity using PLINK? A guide for analyzing medium density SNP data in livestock and pet species

**DOI:** 10.1186/s12864-020-6463-x

**Published:** 2020-01-29

**Authors:** R. Meyermans, W. Gorssen, N. Buys, S. Janssens

**Affiliations:** Department of Biosystems, Livestock Genetics, KU Leuven, Kasteelpark Arenberg 30 – Box 2472, 3001 Leuven, Belgium

**Keywords:** PLINK, Runs of homozygosity, Minor allele frequency, Linkage disequilibrium, SNP density

## Abstract

**Background:**

PLINK is probably the most used program for analyzing SNP genotypes and runs of homozygosity (ROH), both in human and in animal populations. The last decade, ROH analyses have become the state-of-the-art method for inbreeding assessment. In PLINK, the *--homozyg* function is used to perform ROH analyses and relies on several input settings. These settings can have a large impact on the outcome and default values are not always appropriate for medium density SNP array data. Guidelines for a robust and uniform ROH analysis in PLINK using medium density data are lacking, albeit these guidelines are vital for comparing different ROH studies. In this study, 8 populations of different livestock and pet species are used to demonstrate the importance of PLINK input settings. Moreover, the effects of pruning SNPs for low minor allele frequencies and linkage disequilibrium on ROH detection are shown.

**Results:**

We introduce the genome coverage parameter to appropriately estimate F_ROH_ and to check the validity of ROH analyses. The effect of pruning for linkage disequilibrium and low minor allele frequencies on ROH analyses is highly population dependent and such pruning may result in missed ROH. PLINK’s minimal density requirement is crucial for medium density genotypes and if set too low, genome coverage of the ROH analysis is limited. Finally, we provide recommendations for the maximal gap, scanning window length and threshold settings.

**Conclusions:**

In this study, we present guidelines for an adequate and robust ROH analysis in PLINK on medium density SNP data. Furthermore, we advise to report parameter settings in publications, and to validate them prior to analysis. Moreover, we encourage authors to report genome coverage to reflect the ROH analysis’ validity. Implementing these guidelines will substantially improve the overall quality and uniformity of ROH analyses.

## Background

Runs of homozygosity (ROH) are the state-of-the-art method for inbreeding analyses in livestock populations [1]. ROHs are defined as long continuous homozygous stretches in the genome, which are – due to their length – assumed to arise from a common ancestor [2]. Whereas short ROH are indicators of distant inbreeding, long ROH suggest recent inbreeding [3]. ROH were first identified by Broman and Weber in the human genome, whereas Gibson et al. acknowledged their importance for inbreeding calculations [4, 5]. McQuillan et al. defined the genomic inbreeding coefficient based on ROH (F_ROH_) [6].

PLINK [7, 8] is the most used program for ROH analyses in livestock populations [1]. ROH analyses are performed using the *--homozyg* function. The PLINK algorithm for ROH detection relies on a scanning window approach which roughly consists of three steps.

First, the scanning window is defined by a predefined number of SNPs (*--homozyg-window-snp*) with a maximal number of heterozygous SNPs (*--homozyg-window-het*) and a maximal number of missing SNPs (*--homozyg-window-missing*). The defined window stepwise scans an individual’s genome and scores for each SNP the proportion it appears in a homozygous window.

Second, segments of homozygous SNPs are identified genome wide by using a threshold for these scores per SNP: the scanning window hit rate (*--homozyg-window-threshold*). For a window size of 100 SNPs and a threshold of 0.05, a SNP has to appear in at least five homozygous windows before it is identified as part of a segment. Note that such homozygous windows may contain missing or heterozygous SNPs, depending on scanning window settings.

Third, extra constraints are set to these homozygous segments to identify the final ROH segments. The maximal interval between two SNPs in a segment is checked (*--homozyg-gap*) as well as the maximal amount of heterozygous SNPs allowed in the final ROH segment (*--homozyg-het*). Next, ROH segments that do not meet these two requirements are split and re-evaluated. This may lead to detecting ROH segments smaller than the scanning window size. Thereafter, the minimal SNP density (in kb/SNP) per segment is evaluated (*--homozyg-density*) as well as the minimal length and number of SNPs (*--homozyg-kb and --homozyg-snp*). ROH segments which do not fulfill any of these three conditions are removed.

In literature, there is no consensus whether SNP data should be pruned for linkage disequilibrium (LD) and/or minor allele frequency (MAF) before ROH analysis. In Table 1 we provide an overview of recent ROH studies on medium density genotypes using PLINK. Most studies apply MAF pruning with a threshold between 0.01–0.05 and some studies also perform LD pruning. For example, Bjelland et al. and Zhang et al. prune all SNPs with *R*^2^ >  0.5 (using bins of 50 SNPs), resulting in a reduced set of 7997 and 14,366 SNPs (unpruned > 50,000 SNPs), respectively [11, 15]. Hence, this LD pruning results in a SNP reduction of more than 70%.

**Table 1:** Literature review of ROH analysis settings on livestock species using medium density genotypes

Author	Species	LD pruning level (R^2^)	MAF pruning level	--homozyg-window		--homozyg			
				-snp	-threshold	-gap (kb)	-snp	-kb	-density (kb/SNP)
Bosse et al. (2012) [9]	Pig	–	–	20	0.25	–	20	10	1000
Ai et al. (2013) [10]	Pig	–	0.05	50	–	–	–	500	–
Bjelland et al. (2013) [11]	Cattle	> 0.5	0.05	30	–	–	30	–	–
Herrero-Medrano et al. (2013) [12]	Pig	–	–	–	–	1000	20	10	1000
Biscarini et al. (2014) [13]	Cattle	–	–	–	–	1000	–	–	–
Scraggs et al. (2014) [14]	Cattle	–	0.05	50	–	–	50	1000	–
Zhang et al. (2010) [15]	Pig	> 0.5	0.01	–	–	1000	10	5000	500
Al-Mamun et al. (2015) [16]	Sheep	–	0.01	1000	–	250	–	500	–
Mészáros et al. (2015) [17]	Cattle	–	0.01	–	–	–	30	1000	–
Muchadeyi et al. (2015) [18]	Sheep	–	0.05	–	–	500	20	–	50
Rodríguez-Ramilo et al. (2015) [19]	Cattle	–	–	–	–	1000	30	4000	100
Zhang et al. (2015) [20]	Cattle	–	–	20	–	–	–	10	1000
Zanella et al. (2016) [21]	Pig	–	0.03	50	–	–	50	1000	–
François et al. (2017) [22]	Cattle	> 0.5	0.01	50	–	1000	45	500	120
Purfield et al. (2017) [23]	Sheep	–	0.01	50	–	250	–	1000	100
Yang et al. (2017) [24]	Pig	–	0.01	50	–	–	–	500	–
Bortoluzzi et al. (2018) [25]	Chicken	–	0.00^a^	30	–	1000	–	10	1000
Kumar et al. (2018) [26]	Goat	–	0.01	–	–	1000	–	–	70
Mastrangelo et al. (2018) [27]	Sheep	–	0.01	–	–	250	30	1000	100
Michailidou et al. (2018) [28]	Sheep	–	0.01	–	–	–	20	–	–
Zhang et al. (2018) [29]	Pig	–	0.05	50	–	250	L^b^	1000	100
Gorssen et al. (2020) [30]	Pig	–	–	L^b^	–	1000	L^b^	1000	150
Meyermans et al. (2020) [31]	Sheep	–	–	50	0.05	200	L^b^	1000	250

^a^ only monomorphic alleles were deleted from the analysis, ^b^ L parameter as calculated by Lencz et al. [32], −: setting either not performed or not reported in the study

The effect of minimal ROH length, either by the minimal number of SNPs or minimal kb length, has been thoroughly studied by Purfield et al. and Ferenčaković et al. [33, 34]. Purfield et al. concluded that a 50 K SNP array is suitable for identifying ROHs longer than 5 Mb, whereas Ferenčaković et al. reasoned that the minimal ROH length should be adapted to the SNP density. They also found that heterozygous calls should be tolerated depending on the ROH length and SNP density [34]. Note that when allowing more than one heterozygous SNP in a scanning window, adjacent heterozygous SNPs may cause the merging of different homozygous segments which are longer than the original ones.

Howrigan et al. simulated genotypes to test PLINK’s ROH detection ability and varied several PLINK detection settings (*--homozyg-window-snp*, *−-homozyg-window-het*, *--homozyg-window-missing*, *--homozyg-window-threshold*, *--homozyg-snp*) [35]. They concluded that data should be pruned for LD and MAF prior to analysis. However, Howrigan and colleagues did not vary scanning window sizes, maximal gap sizes, minimal density requirements (in kb/SNP) nor final ROH length in kb, although these parameters can affect the outcome [35].

There is a large variation in parameter settings considering the maximal gap, minimal density and the scanning window size (Table 1). Moreover, studies often do not report density, gap and/or window size settings. Both Howrigan et al. and Peripolli et al. underlined a lack of consensus criteria for ROH analyses [1, 35]. This lack of consensus will lead to biased results and hinders the comparison of results across studies.

In this paper, we provide guidelines for choosing PLINK parameter settings that ensure a robust and reliable ROH analysis. We used medium density genotypes in eight different livestock and pet species (pig, cattle, sheep, cats, horses, goats, dogs and chicken). First, we evaluated the effect of MAF and LD pruning on ROH analysis. Second, we investigated effects of the minimal density (*--homozyg-density*), the maximal interval between two SNPs in a ROH (*--homozyg-gap*), scanning window size (*--homozyg-window-snp*) and scanning window hit rate (*--homozyg-window-threshold*). Third, we introduce the genome coverage parameter to evaluate the validity of the ROH analysis and to estimate inbreeding based on ROH more accurately. These guidelines facilitate an adequate and robust ROH analysis, resulting in a higher overall quality and uniformity across studies.

## Results

All analyses were performed on the eight different livestock and pet breeds. Results and figures for PIT, BB, MER and BUR are provided in the main manuscript, whereas results for SAA, ICE, LAB and BAR can be found in Additional files 1, 2, 3, 4, 5, 6 and 7.

### Pruning for linkage disequilibrium

**Fig. 1:**
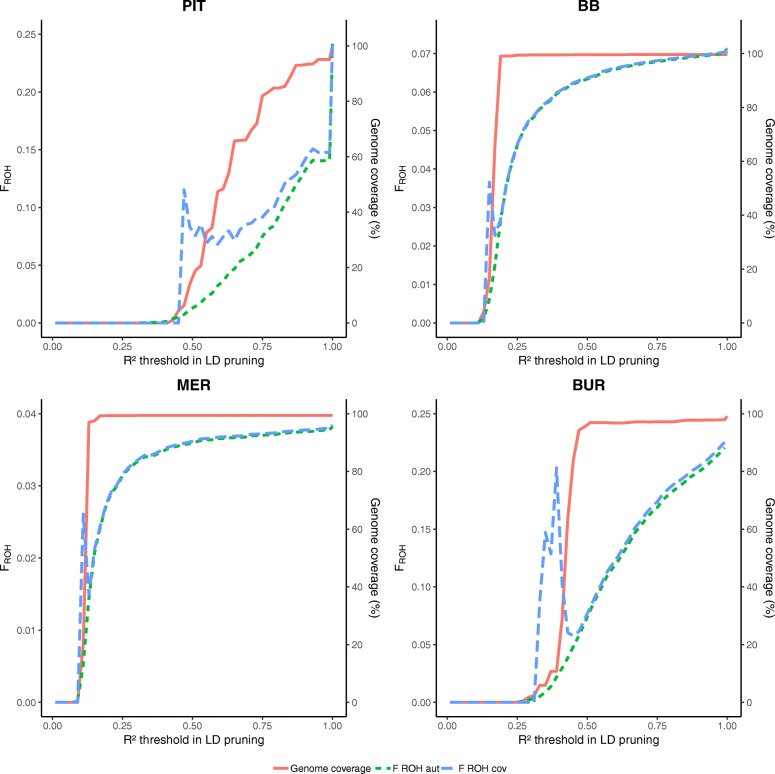
Pruning for linkage disequilibrium (LD) has an impact on genome coverage and FROH estimation in PLINK. Genome coverage and estimated F_ROH cov_ and F_ROH aut_ are shown for different levels of LD pruning in Pietrain pigs (PIT), Belgian Blue cattle (BB), Australian Polled Merino sheep (MER) and Burmese cats (BUR). R^2^ values show the threshold values at which pruning was performed (SNPs with R^2^ > threshold were pruned from the dataset). R^2^ equals 1 when not pruned for LD

The results of pruning for varying LD levels prior to ROH analysis for PIT, BB, MER and BUR are shown in Fig. 1, results for SAA, ICE, LAB and BAR are added in Additional file 2: Figure S1. The effects of LD pruning on the outcome of the ROH analysis was population dependent. Although maximal genome coverage was reached at *R*^2^ >  0.25 in some populations (e.g. BB), not all ROH were detected and F_ROH_ estimates were lower than without pruning for LD. In PIT, maximal genome coverage was reached more slowly in comparison to other populations (e.g. BB).

### Pruning for minor allele frequency

**Fig. 2:**
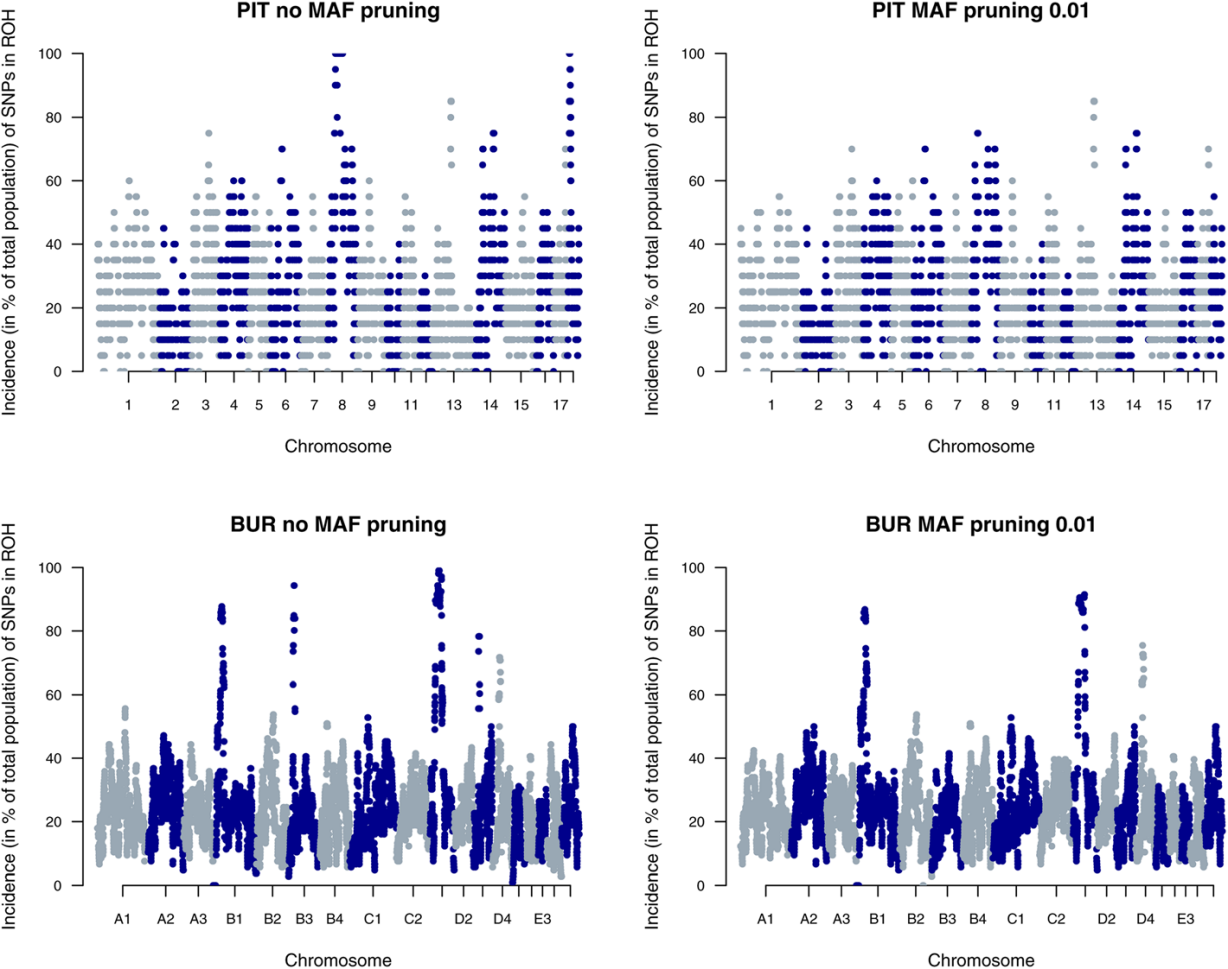
Incidence plots of SNPs in a ROH for Pietrain pigs (PIT) and Burmese cats (BUR) in PLINK. Results show that pruning data for MAF (0.01) may result in a decreased ROH detection, especially in highly homozygous regions (e.g. SSC8 and SSC18 in PIT)

In PIT and BUR, we observed that even mild MAF pruning (0.01) had an impact on ROH detection in several genomic regions. Figure 2 shows ROH incidence per SNP (in % of the total population) for both populations without MAF pruning (left) and with MAF pruning at 0.01 (right). For PIT, ROH islands were observed on SSC8 and SSC18, whereas for BUR, a change in observed ROH was found on e.g. B3, D1 and D3. These ROH in PIT and BUR would not have been detected if MAF pruning was performed. For the six other populations, little differences were observed in genome coverage and F_ROH_ estimates by varying MAF pruning levels.

### Minimal density requirement

**Fig. 3:**
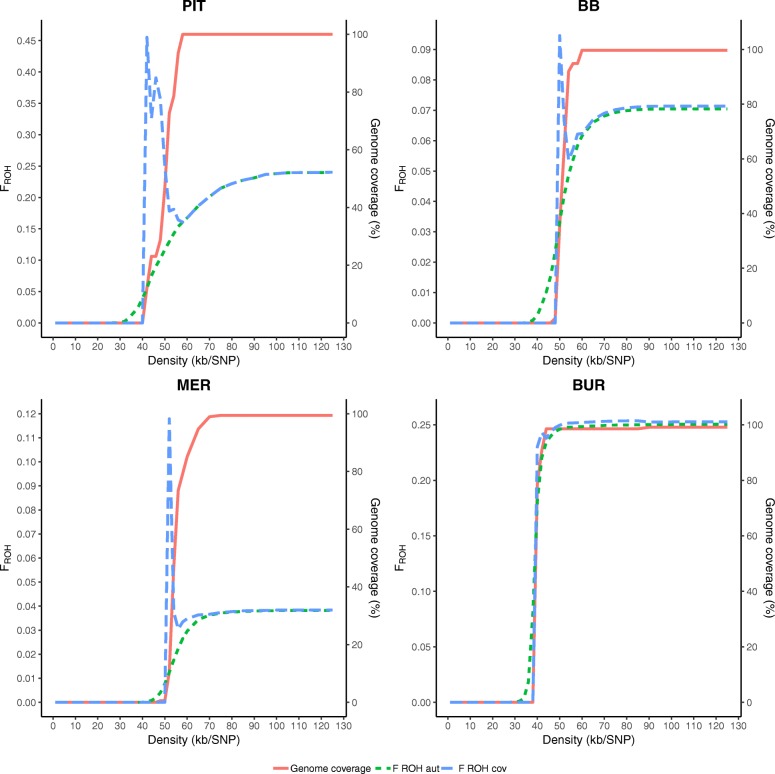
Settings of the minimal density requirement in ROH analyses in Plink may influence the outcome. Genome coverage, estimated F_ROH cov_ and F_ROH aut_ are showed for varying density from 1 to 125 kb/SNP for Pietrain pigs (PIT), Belgian Blue cattle (BB), Australian Polled Merino sheep (MER) and Burmese cats (BUR

Figure 3 presents the genome coverage (in %) and the estimated F_ROH, aut_ and F_ROH, cov_ by varying density for PIT, BB, MER and BUR (results for SAA, ICE, LAB and BAR are shown in Additional file 3: Figure S2). All investigated populations showed a low genome coverage with density below 40 kb/SNP. Starting from a mean density of 40 kb/SNP genome coverage increased and maximal coverage is reached between 60 and 75 kb/SNP.

### Maximal gap requirement

**Fig. 4:**
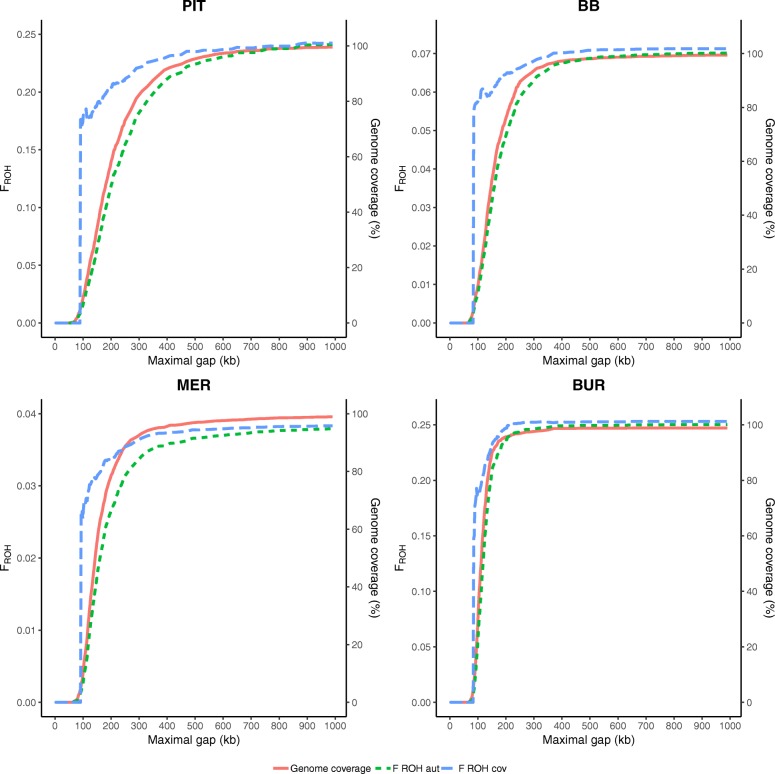
Settings of the maximal gap requirement in ROH analyses in PLINK may influence the outcome. This figure shows the genome coverage, estimated F_ROH cov_ and F_ROH aut_ for varying gaps from 1 to 1000 kb for Pietrain pigs (PIT), Belgian Blue cattle (BB), Australian Polled Merino sheep (MER) and Burmese cats (BUR)

The results for varying maximal gap settings in ROH analyses for PIT, BB, MER and BUR are shown in Fig. 4, results for SAA, ICE, LAB and BAR are added in Additional file 4: Figure S3. All investigated populations reached maximal genome coverage using gap sizes around 500 kb. Below 500 kb, genome coverage decreased as well as F_ROH cov_ estimates. In general, F_ROH aut_ decreased faster than F_ROH cov_.

### Scanning window size and threshold

An increasing scanning window size led to a decrease in estimated F_ROH_, where especially short ROH were no longer detected. Similarly, an increasing threshold resulted in a decreasing F_ROH_. For both settings, genome coverage did not vary. Results are shown in Additional file 5: Figure S4 and Additional file 6: Figure S5.

### Validation using a model based approach for ROH detection

**Fig. 5:**
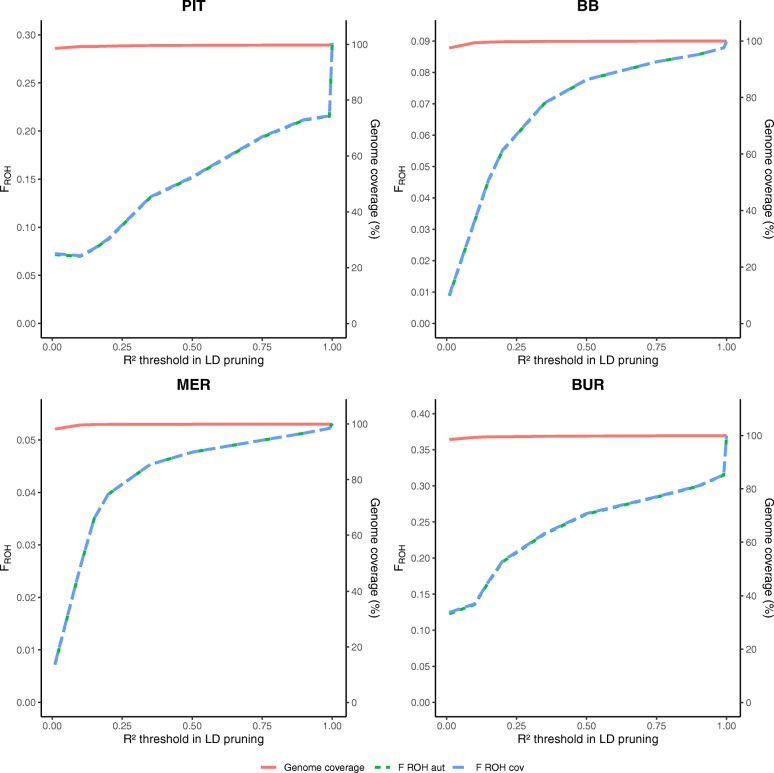
Pruning for linkage disequilibrium (LD) has an impact on genome coverage and FROH estimation in RZooRoH. Genome coverage and estimated F_ROH cov_ and F_ROH aut_ are shown for different levels of LD pruning in Pietrain pigs (PIT), Belgian Blue cattle (BB), Australian Polled Merino sheep (MER) and Burmese cats (BUR). *R*^2^ values show the threshold values at which pruning was performed (SNPs with *R*^2^ > threshold were pruned from the dataset). *R*^2^ equals 1 when not pruned for LD

In general, the model based approach (RZooRoH) yielded higher F_ROH_ estimates than the rule based approach (PLINK) (Fig. 1 vs Fig. 5). This can be attributed to the less stringent constraints of the model based approach (e.g. no minimal ROH length). Pearson correlations of individual F_ROH_ between PLINK and RZooRoH were high (*r* = 0.89–0.99) for all populations (no LD nor MAF pruning performed).

**Fig. 6:**
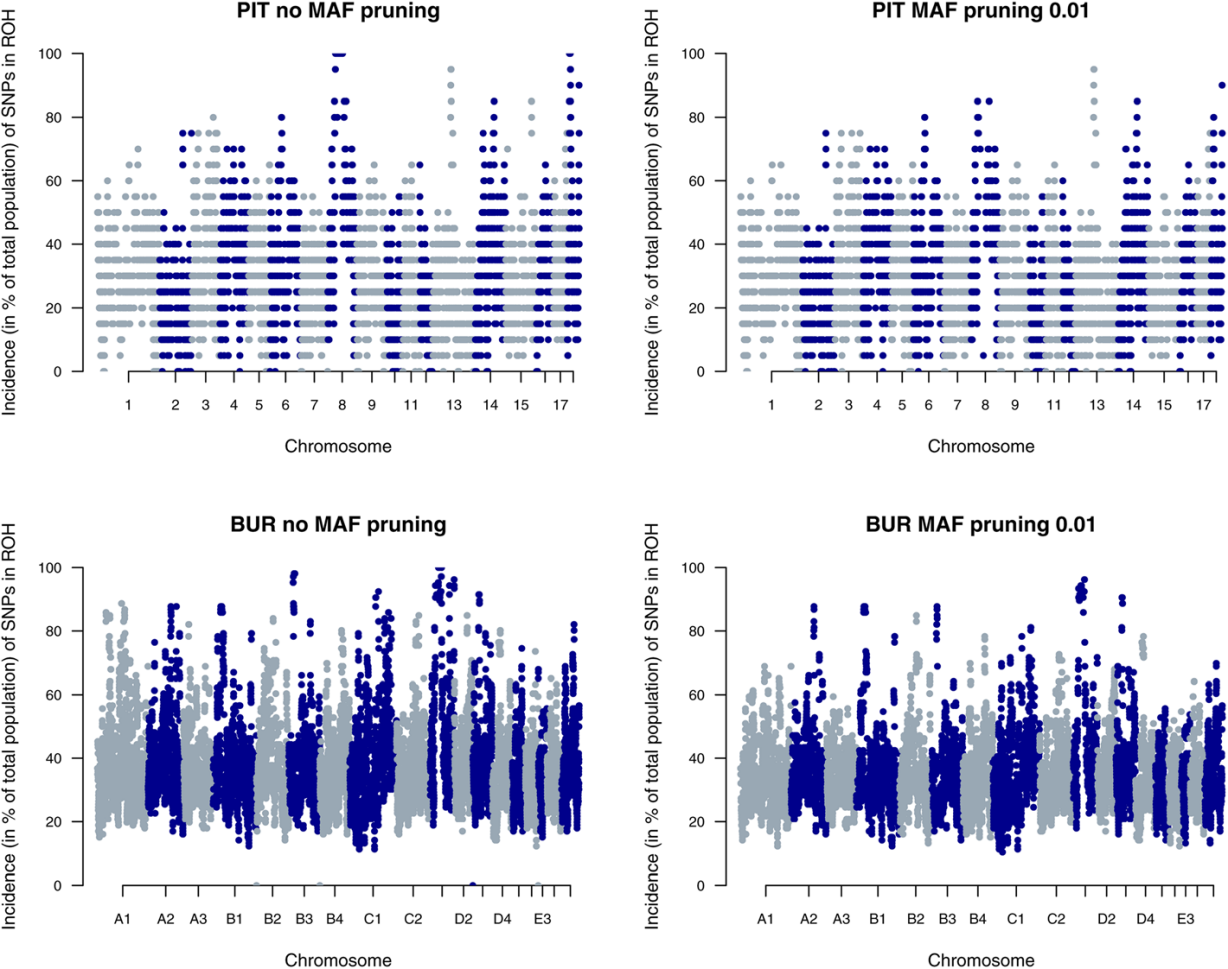
Incidence plots of SNPs in a ROH for Pietrain pigs (PIT) and Burmese cats (BUR) in RZooRoH. Results show that pruning data for MAF (0.01) may result in a decreased ROH detection, especially in highly homozygous regions (e.g. SSC8 and SSC18 in PIT)

Results for varying LD levels prior to ROH analysis using a model based approach (RZooRoH) for PIT, BB, MER and BUR are shown in Fig. 5, while results for SAA, ICE, LAB and BAR are added in Additional file 7: Figure S6. MAF pruning using RZooRoH revealed the same results: in PIT and BUR, the same effects of even mild MAF pruning (0.01) on ROH detection were observed (Fig. 2 vs Fig. 6), whereas in the other six populations no substantial differences were apparent.

## Discussion

To unravel the effects of PLINK parameter settings on ROH estimation using medium density SNP data we analyzed these settings on eight different livestock and pet species. We examined the effects of pruning for LD and/or MAF on ROH detection and genome coverage. Next, we investigated the effect of the previously unstudied PLINK parameters.

### Pruning for linkage disequilibrium

The effect of LD pruning on ROH analysis is highly population dependent (Fig. 1 and Additional file 2: Figure S1). For PIT, genome coverage quickly drops with an increased level of LD pruning (e.g. for *R*^2^ >  0.5, genome coverage is only 16.3%). For PIT and BUR, F_ROH_ shows a strong decrease for more stringent LD conditions, whereas in BB, MER and SAA this strong decrease could not be observed.

Howrigan et al. suggested to perform LD pruning before ROH analysis, based on their study using simulated genotypes [35]. However, we showed that LD pruning can have major effects on the ROH analysis when working with real genotypes from sampled populations. The main reason for LD pruning is to exclude short and common segments that can be assigned as ROH but which are more likely to have arisen from LD [33, 34, 36]. However, we showed that LD pruning also affects the detection of large ROH. Furthermore, LD patterns are highly dependent on population structure [37]. In inbred populations, pruning SNP for high LD leads to a severe reduction of SNP density in homozygous genomic regions, leading to a biased ROH analysis. Validation using a model based approach confirmed that LD pruning has a population dependent effect on ROH detection (Fig. 5 and Additional file 7: Figure S6). For BAR, at unrealistically high LD pruning levels (*R*^2^ >  0.35), deviant results were observed possibly due to the high inbreeding level, presence of microsomes and the small chicken genome.

Therefore, we argue that caution has to be taken in performing LD pruning prior to ROH analysis, due to differences in population structure. We suggest to correct for false positive ROH, caused by LD, by increasing the stringency criteria for ROH detection (e.g. minimal ROH length in number of SNP and kb) instead of pruning SNPs for LD, as previously reported [33, 34].

### Pruning for minor allele frequency

In literature, there is no consensus whether or not genotypes should be pruned for low MAF before ROH analysis. Our inventory of recent studies in livestock (Table 1) showed that most studies prune data for MAF < 0.01 or < 0.05. Howrigan et al. [35] recommended to prune genotypes for low MAF based on their study using simulated genotypes. However, for both methods (PLINK and RZooRoH) we showed that pruning for low MAF can ignore large homozygous regions in the genome (e.g. in PIT and BUR) (Figs. 2 and 6). For PIT, MAF pruning of 0.01 overlooked large homozygous regions on SSC8 (length ± 50 Mb) and on SSC18 (length ± 10 Mb). These regions also appeared nearly fixed in a study on 1632 Pietrains [30]. For BUR, ROH went undetected on e.g. B3, D1 and D3 due to pruning for MAF. Not only ROH detection on fixed regions was affected by MAF pruning (e.g. D3 in BUR), but also in non-fixed regions (e.g on. B3 and D3 in BUR) large differences in ROH incidence were detected.

Pruning for low MAF as a quality control measure was introduced in genome wide association studies (GWAS) for two main reasons. First, genotype accuracy declines with decreasing MAF [38]. Second, the detection probability for an association decreases with lower MAF, making SNPs with low MAF less important for GWAS [38]. However, in contrast to GWAS, ROH analyses do not aim to find an association between individual SNPs and phenotype, but examine homozygous stretches of multiple SNPs. Since multiple SNPs in a sequence are evaluated, a decreased genotyping accuracy for a single SNP will have a minimal effect on the ROH analysis. Moreover, MAF pruning did not affect F_ROH_ estimates and genome coverage in other populations (e.g. BB), indicating that MAF pruning does not improve ROH detection. Therefore, we recommend not to perform MAF pruning before ROH analysis.

### Minimal density requirement

We showed that the minimal density setting (in kb/SNP) can strongly affect the outcome of the ROH analysis. This is most relevant for medium density SNP arrays since in PLINK the default density setting is roughly equal to the average density for most livestock and pet species (50 kb/SNP). This is clearly visible in Fig. 3, where genome coverage and F_ROH_ sharply increase between 40 and 60 kb/SNP. At the default PLINK setting (50 kb/SNP), genome coverage in PIT was only 47% and for MER barely 0.6%. Calculating the genome coverage parameter proves to be a good method to check whether the density parameter is set appropriately. For the studied populations maximal genome coverage was reached between 60 and 70 kb/SNP.

Moreover, SNP densities can differ along the genome (e.g. > 150 kb/SNP on SSC3 in PIT) and therefore certain regions could be overlooked. A possible solution is to implement a check for density on the scanning window level in PLINK’s algorithm (implement *density* as *--homozyg-window-density* instead of *--homozyg-density*). This forces the algorithm to check every window for the required minimal density instead of only performing the check on (large) homozygous regions.

Note that the average chicken genome length (±1000 Mb) is about half the size of the average mammalian genome, and therefore the minimal density at which maximal genome coverage is reached lies around 25 kb/SNP (e.g. BAR, Additional file 3: Figure S2). Furthermore, LAB was genotyped on an 110 K array and thus the average SNP density was twice as dense as the other populations, causing maximal genome coverage to be reached at ±20 kb/SNP (Additional file 3: Figure S2).

### Maximal gap requirement

A wide variety of gap settings is reported in literature and little motivation is given for their use (Table 1). For all studied populations, the default PLINK gap (1000 kb) covers over 99% of the detectable autosome (Fig. 4). Only for gap sizes below 500 kb, genome coverage drops below 95%. The default value of 1000 kb is therefore set too high to influence ROH detection even for medium density data. To determine the optimal gap setting, high density genotypes or sequence data are necessary. This way, one can determine at which maximal gap it is no longer safe to assume that underlying SNPs are homozygous. Anticipating further research, we suggest to minimize gap length while maintaining maximal genome coverage and we advise to use F_ROH cov_ for inbreeding estimation.

### Scanning window size and threshold

With an increasing window size and window threshold, F_ROH_ decreases due to more stringent conditions to identify a homozygous segment. We recommend choosing the scanning window size parameter equal to *L*, the minimal length of a ROH. On the one hand, setting the scanning window size below *L* will not result in the detection of new ROH, as the minimal length of a ROH is set at *L*. On the other hand, a window size higher than *L* SNPs prohibits the detection of ROH with a minimal size of *L* SNPs, as Curik et al. also pointed out [3].

The scanning window threshold affects the number of outer SNPs in a homozygous segment that cannot be included reliably in the final ROH segment. After all, it is possible that outer SNPs of the homozygous segment are homozygous rather by chance than by descent.

We propose to calculate the scanning window threshold (*t*) as follows:

$$ t= floor\left(\frac{N_{out}+1}{L},3\right) $$

with N_out_ the desired number of final outer SNPs on either side of the homozygous segment that should not be included in the final ROH and *L* the scanning window size. In this formula, ‘*+ 1’* is included as this denotes the first SNP that will be tolerated of the final ROH and ‘*, 3*’ points at flooring with three decimals. For example, with *L* = 100 and N_out_ = 4, the threshold will be set at 0.05. By doing this, we will scan windows of 100 SNPs and in the obtained homozygous segment we discard the four outer SNPs on each side of the homozygous segment.

Most studies do not report the scanning window threshold setting, although it impacts the outcome of ROH analysis. Therefore, we encourage authors to always specify the scanning window threshold.

### Comparison with literature

This study shows that PLINK settings and pruning for either LD or MAF are extremely important for ROH analysis outcome. Nevertheless, as Table 1 shows, several parameter settings are often not explicitly mentioned. Therefore it is unclear whether some settings were left unadjusted or ignored. Ten out of 23 studies in Table 1 used a density of 50 kb/SNP or did not mention a change of the density setting. It is possible that genome coverage in these studies is strongly reduced and consequently, F_ROH_ could be underestimated for these studies.

As an example, we evaluated the genome coverage of the ROH analysis as performed by Yang et al. on 146 pig populations [24]. We found a genome coverage of only 34.2%, mainly due to MAF pruning and use of the unadapted default density parameter [24]. Therefore, the F_ROH_ estimate for PIT was equal to 7.6% (using the F_ROH_ calculation method of McQuillan et al. [6]), whereas we estimated the average F_ROH_ for PIT to be at least a threefold higher. However, the spearman rank correlation on population level for all 146 populations between our estimated F_ROH cov_ and the average F_ROH_ reported by Yang et al. was high (*r* = 0.90).

## Conclusions

This study has shown that MAF and LD pruning as well as PLINK input settings can severely impact ROH analyses on medium density genotypes.

Pruning for low MAF and LD was historically introduced in genomic analyses but seems to provide little benefits for ROH studies. Our findings show that MAF and LD pruning can be problematic for ROH detection, regardless of the method used (rule based or model based). Therefore we recommend to skip MAF and LD pruning prior to all ROH analyses using medium density genotypes.

It is clear from our results that a low minimal density setting (in kb/SNP) can lead to an incomplete genome coverage of the analysis and should be evaluated thoroughly. Moreover, the default PLINK setting of 50 kb/SNP is often not suitable. Furthermore, we advise to minimize the maximal gap setting while still assuring maximal genome coverage. The scanning window size should be kept equal to the minimal desired ROH length. Finally, a proposal to calculate the scanning window threshold is given.

Overall, we advocate to always report PLINK input settings in publications, and to validate them prior to analysis. Moreover, we encourage to use and report genome coverage to reflect the ROH analysis’ validity. Based on this genome coverage, F_ROH_ can be more accurately estimated. We strongly believe that using these recommendations will improve quality and comparability of ROH analyses.

## Material and methods

The effect of LD and MAF pruning on ROH detection, as well as the effects of the density, gap and window size settings were evaluated on different livestock and pet species (pig, sheep, horse, cattle, goat, chicken, cat and dog). We demonstrated our findings in one example population per species (Table 2).

**Table 2:** Overview of the selected populations per species

Species	Population	Abbreviation	N	Source
Pig	Pietrain	PIT	20	[24]
Cattle	Belgian Blue	BB	766	Own data
Sheep	Australian Polled Merino	MER	98	[39]
Horse	Swedish bred Icelandic	ICE	209	[40]
Goat	Saanen	SAA	171	[41]
Chicken	Barnevelder	BAR	24	[25]
Cat	Burmese	BUR	106	[42]
Dog	Labrador	LAB	728	[43]

All populations were genotyped on medium density arrays. We performed our analyses on the autosomal genome, discarding all SNPs with unassigned chromosomal information and all SNPs with a low call rate (< 95%). Detailed information on the quality control for all populations is given in Additional file 1: Table S1.

Many studies estimate the inbreeding coefficient based on ROH (F_ROH_) as [6]:

$$ {F}_{ROH}=\frac{L_{ROH}}{L_{aut}}, $$

where *L*_*ROH*_ is the total length of all ROHs in the individual’s genome, and *L*_*aut*_ is the length of the autosomal genome. The population’s mean F_ROH_ is calculated as the average F_ROH_ of all individuals. However, the length of the autosomal genome depends on the genome assembly used for SNP mapping and can therefore differ between genotyping arrays. Moreover, regional differences in SNP density can result in genomic regions where it is impossible to detect ROH. The detection of ROH in these regions is not only dependent on the SNP density of the array, but also on the specific criteria assigned to PLINK to detect ROH.

Therefore, we propose two different methods for estimating F_ROH_. First F_ROH, aut_ is the estimated degree of inbreeding based on the length of the autosomal genome, with *L*_*aut*_ calculated as the length between the first SNP and the last SNP per chromosome for all autosomal chromosomes. Second, F_ROH, cov_ is the estimated degree of inbreeding based on the length of the covered genome, where *L*_*aut*_ is equal to the length of the autosomal genome where ROH detection is possible. This was calculated by simulating an individual with a completely homozygous genotype (based on the population’s *.map* file) and performing the ROH analysis with all specified parameters on this homozygous individual. The total ROH length found for this homozygous individual is the maximal detectable ROH length for any individual in this population, given the parameter settings. Similarly, we calculated the genome coverage of the ROH analysis as the proportion of the maximal detectable ROH length over the length of the (autosomal) genome. This genome coverage was as an indication of the validity of the ROH analysis.

To study the effect of MAF pruning, we used PLINK’s *--maf* function for MAF equal to 0.01, 0.05, 0.10 and 0.20 and compared this to the ROH analysis without MAF pruning. To analyze the effect of LD pruning on ROH analyses, we used PLINK’s *--indep-pairwise* function with a scanning window of 50 (step size of 5) and pruned SNPs with R^2^ values between 0.01 and 0.99. These results were compared to the ROH analysis without LD pruning (*R*^2^ = 1). To test the minimal density setting, we varied --homozyg-density from 10 to 125 kb/SNP. To examine the effect of the maximal gap setting, we varied *--homozyg-gap* from 1 to 1000 kb. The scanning window size setting (*--homozyg-window-snp*) was investigated by varying this setting from 1 to 150 SNPs. The scanning window threshold (*--homozyg-window-threshold*) was varied between 0.05 and 0.95. The PLINK settings were evaluated on non-pruned genotypes. When unvaried, ROH detection settings were set to a small scanning window (20 SNPs), a large gap (2 Mb), a high density level (200 kb/SNP) and a scanning window threshold level of 0.05. All ROH detection was performed with a minimal ROH length of 1 Mb, maximum one missing SNP and no heterozygous SNPs in the scanning window. The minimal number of SNPs in a ROH was determined by the formula proposed by Lencz et al. and adapted by Purfield et al. [32, 33]:

$$ L=\frac{lo{g}_e\frac{\alpha }{n_s{n}_i}}{lo{g}_e\left(1- het\right)}, $$

with *n*_*s*_ the number of genotyped SNPs per individual, *n*_*i*_ the number of genotyped individuals, *α* the percentage of false positive ROH (0.05) and *het* the mean heterozygosity across all SNPs.

We validated our results using PLINK with a non-rule based ROH detection method by analyzing all populations using the model based software RZooRoH, developed by Druet and Gauthier [44]. The RZooRoH software identifies homozygous-by-descent (HBD) segments associated with ROHs and is based on a hidden Markov model framework. We used a two-states model (1R model), which estimates the probability between two consecutive markers to be either HBD or non-HBD [44]. The genotyping error rate was set to 0.25%, as suggested by Ferenčaković et al. [34]. Furthermore, allele frequencies of reference populations were provided to the algorithm, as suggested by Dr. Tom Druet (personal communication). To compare F_ROH_ results from both PLINK and RZooRoH, an individual with a completely homozygous genotype was simulated, using the same method as previously described. This individual was analyzed in RZooRoH to calculate the maximal detectable ROH length for any individual in the population, given the model settings.

## Supplementary information

**Additional file 1: Table S1.** Quality control metrics for all evaluated populations. Abbreviations as in Table 2.

**Additional file 2: Figure S1.** The effect of linkage disequilibrium (LD) pruning on genome coverage and F_ROH_ estimates for ICE, SAA, LAB and BAR in PLINK.

**Additional file 3: Figure S2.** The effect of the density setting (in kb/SNP) on genome coverage and F_ROH_ estimates for ICE, SAA, LAB and BAR.

**Additional file 4: Figure S3.** The effect of maximal gap setting (in kb) on genome coverage and F_ROH_ estimates for ICE, SAA, LAB and BAR.

**Additional file 5: Figure S4.** The effect the scanning window size on genome coverage and F_ROH_ estimates for all evaluated populations.

**Additional file 6: Figure S5.** The effect of the scanning window threshold on genome coverage and F_ROH_ estimates for all evaluated populations.

**Additional file 7: Figure S6.** The effect of linkage disequilibrium (LD) pruning on genome coverage and F_ROH_ estimates for ICE, SAA, LAB and BAR in RZooRoH. For BAR, an increase in F_ROH_ was detected at very low *R*^2^ values (> 0.35), probably linked to a high degree of inbreeding and a strong decrease in number of markers in a small genome to reliably estimate HBD.

## Data Availability

The dataset of Belgian Blue genotypes used in the current study are accessible via Figshare [DOI: 10.6084/m9.figshare.11441283]. The online datasets are available at: • Bortoluzzi C, Crooijmans RPMA, Bosse M, Hiemstra SJ, Groenen MAM, Megens H (2018) Data from: The effects of recent changes in breeding preferences on maintaining traditional Dutch chicken genomic diversity. Dryad Digital Repository. 10.5061/dryad.1d832h3 • Colli L, Milanesi M, Talenti A, Bertolini F, Chen M, Crisà A, Daly K, Del Corvo M, Guldbrandtsen B, Lenstra JA, Rosen BD, Vajana E, Catillo G, Joost S, Nicolazzi EL, Rochat E, Rothschild MF, Servin B, Sonstegard TS, Steri R, Van Tassel CP, Ajmone-Marsan P, Crepaldi P, Stella A, AdaptMap Consortium (2018) Data from: Signatures of selection and environmental adaptation across the goat genome post-domestication. Dryad Digital Repository. 10.5061/dryad.v8g21pt • Gandolfi, B., Alhaddad, H., Abdi, M., Bach, L. H., Creighton, E. K., Davis, B. W., … Lyons, L. A. (2018). Applications and efficiencies of the first cat 63 K DNA array. *Scientific Reports*, *8*(1), 7024. Data available on: https://www.nature.com/articles/s41598-018-25438-0#Sec26 • Kijas, J. W., Lenstra, J. A., Hayes, B., Boitard, S., & Neto, P. (2012). Genome-Wide Analysis of the World’s Sheep Breeds Reveals High Levels of Historic Mixture and Strong Recent Selection. *PLoS Biology*, *10*(2), 1001258. Data available on: http://www.sheephapmap.org/download.php • Shrestha M, Eriksson S, Schurink A, Andersson LS, Sundquist M, Frey R, Broström H, Bergström T, Ducro B, Lindgren G (2015) Data from: Genome-wide association study of insect bite hypersensitivity in Swedish-born Icelandic horses. Dryad Digital Repository. 10.5061/dryad.9r161.2 • Wiener P, Sánchez-Molano E, Clements DN, Woolliams JA, Haskell MJ, Blott SC (2017) Data from: Genomic data illuminates demography, genetic structure and selection of a popular dog breed. Dryad Digital Repository. 10.5061/dryad.38q43 • Yang B, Cui L, Perez-Enciso M, Traspov A, Crooijmans RPMA, Zinovieva N, Schook LB, Archibald A, Gatphayak K, Knorr C, Triantafyllidis A, Alexandri P, Semiadi G, Hanotte O, Dias D, Dovč P, Uimari P, Iacolina L, Scandura M, Groenen MAM, Huang L, Megens H (2017) Data from: Genome-wide SNP data unveils the globalization of domesticated pigs. Dryad Digital Repository. 10.5061/dryad.30tk6

## Abbreviations

BAR: Barnevelder chicken population
BB: Belgian Blue cattle population
BUR: Burmese cat population
F_ROH aut_: Genomic ROH inbreeding coefficient (calculation based on autosomal genome length)
F_ROH cov_: Genomic ROH inbreeding coefficient (calculation based on the genome coverage)
F_ROH_: Genomic inbreeding coefficient based on ROH
GWAS: Genome wide association study
HBD: Homozygous-by-descent
het: Mean heterozygosity across all SNPs
ICE: Swedish bred Icelandic horse population
kb: Kilo base pairs
L: Minimal required ROH length in SNPs
LAB: Labrador dog population
L_aut_: Length of the autosomal genome
LD: Linkage disequilibrium
L_ROH_: Length of all ROHs in an individual’s genome
MAF: Minor allele frequency
Mb: Mega base pairs
MER: Australian polled Merino sheep population
n_i_: Number of genotyped individuals
N_out_: Desired number of final outer SNPs on either side of the homozygous segment that should not be included in the final ROH
n_s_: Number of genotyped SNPs per individual
PIT: Pietrain pig population
R^2^: Inter-variant allele count squared correlations
ROH: Run of homozygosity
SAA: Saanen goat population
SNP: Single nucleotide polymorphism
t: Threshold of PLINK’s scanning window
α: Percentage of false positive ROH (0.05)
