# Supplementary material for: How to study runs of homozygosity using PLINK? A guide for analyzing medium density SNP data in livestock and pet species
Source: BMC Genomics. 2020 Jan 29;21:94. doi: 10.1186/s12864-020-6463-x (PMC6990544; doi:10.1186/s12864-020-6463-x)
Supplement: Supplementary file 1 — Additional file 1: Table S1. Quality control metrics for all evaluated populations. Abbreviations as in Table 2. [file 12864_2020_6463_MOESM1_ESM.docx]

**Table S1. Quality control metrics for all evaluated populations.** Abbreviations as in Table

|  | PIT | BB | MER | ICE | SAA | BUR | LAB | BAR |
| --- | --- | --- | --- | --- | --- | --- | --- | --- |
| Number of genotyped animals | 20 | 766 | 98 | 209 | 171 | 106 | 728 | 24 |
| Total number of SNPs before quality control | 61772 | 50908 | 49034 | 54602 | 53347 | 62272 | 117971 | 52232 |
| No chromosomal coordinates | 7576 | 536 | 110 | 0 | 1407 | 0 | 0* | 556 |
| SNPs on sex chromosomes and mitochondria | 1413 | 1139 | 1341 | 2539 | 1987 | 3384 | 0* | 2584 |
| Call rate (<0.95) | 4998 | 551 | 48 | 1914 | 2242 | 2228 | 0* | 4643 |
| Total removed SNPs | 13987 | 2226 | 1499 | 4453 | 5636 | 5612 | 0* | 7783 |
| Total SNPs passing quality control | 47785 | 48682 | 47693 | 50149 | 47711 | 56660 | 117971 | 44449 |

*: available data were already submitted to quality control
