# Supplementary figures and images for: How to study runs of homozygosity using PLINK? A guide for analyzing medium density SNP data in livestock and pet species
Source: BMC Genomics. 2020 Jan 29;21:94. doi: 10.1186/s12864-020-6463-x (PMC6990544; doi:10.1186/s12864-020-6463-x)

ICE

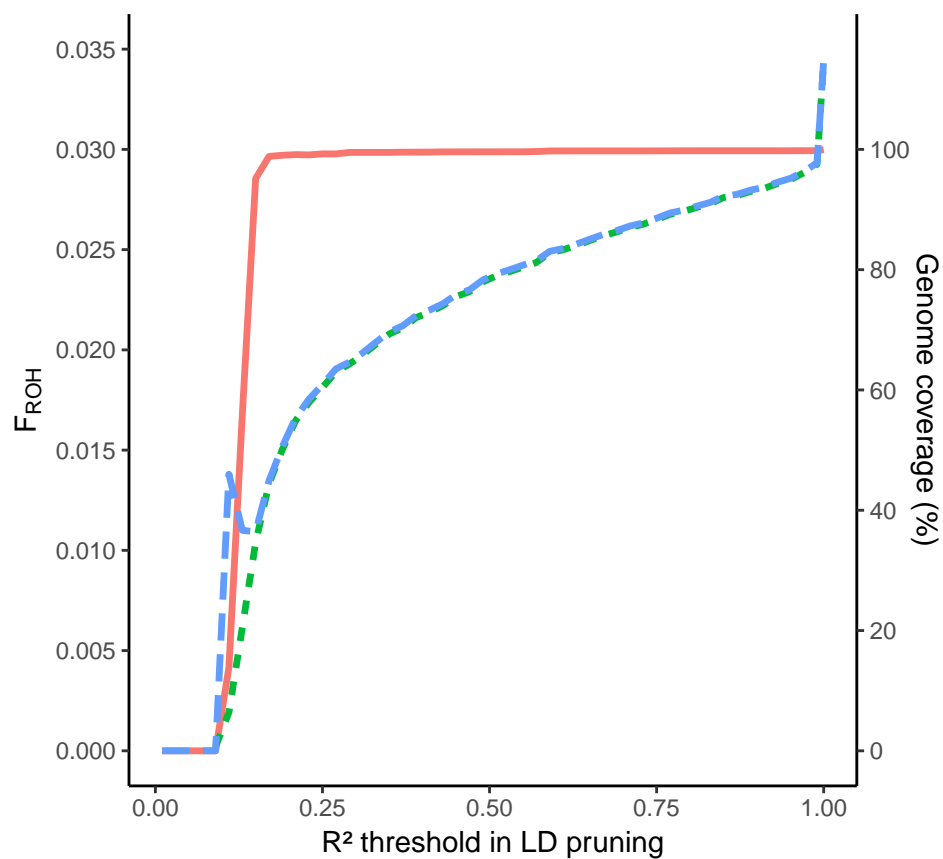

SAA

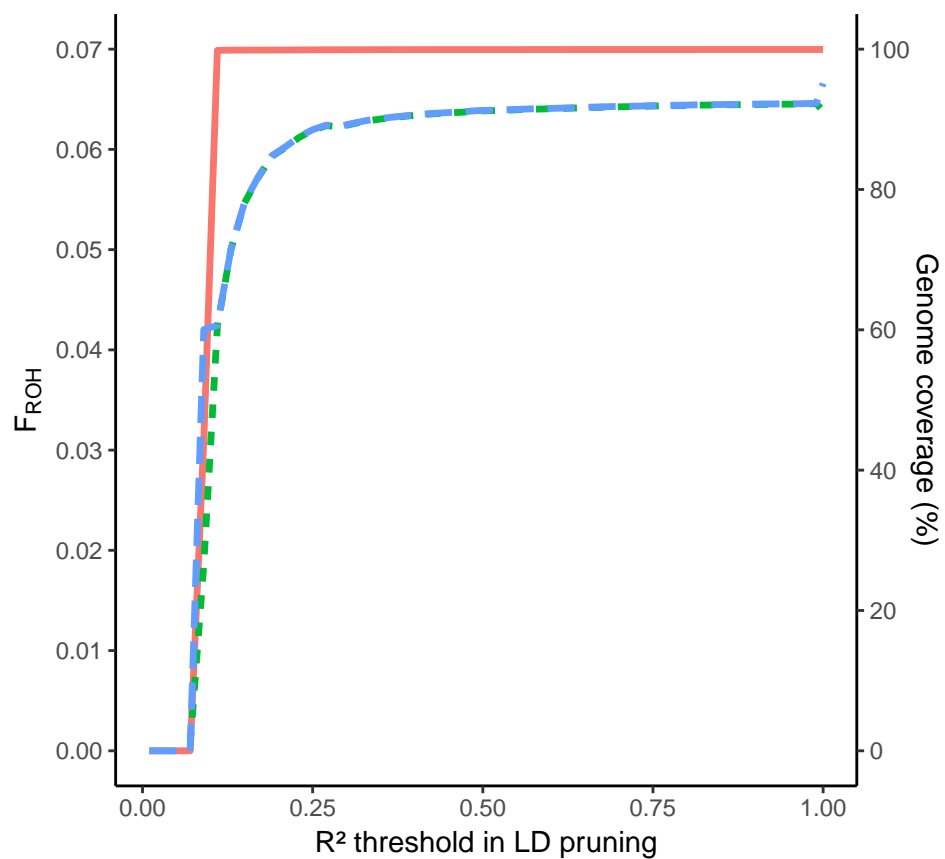

LAB

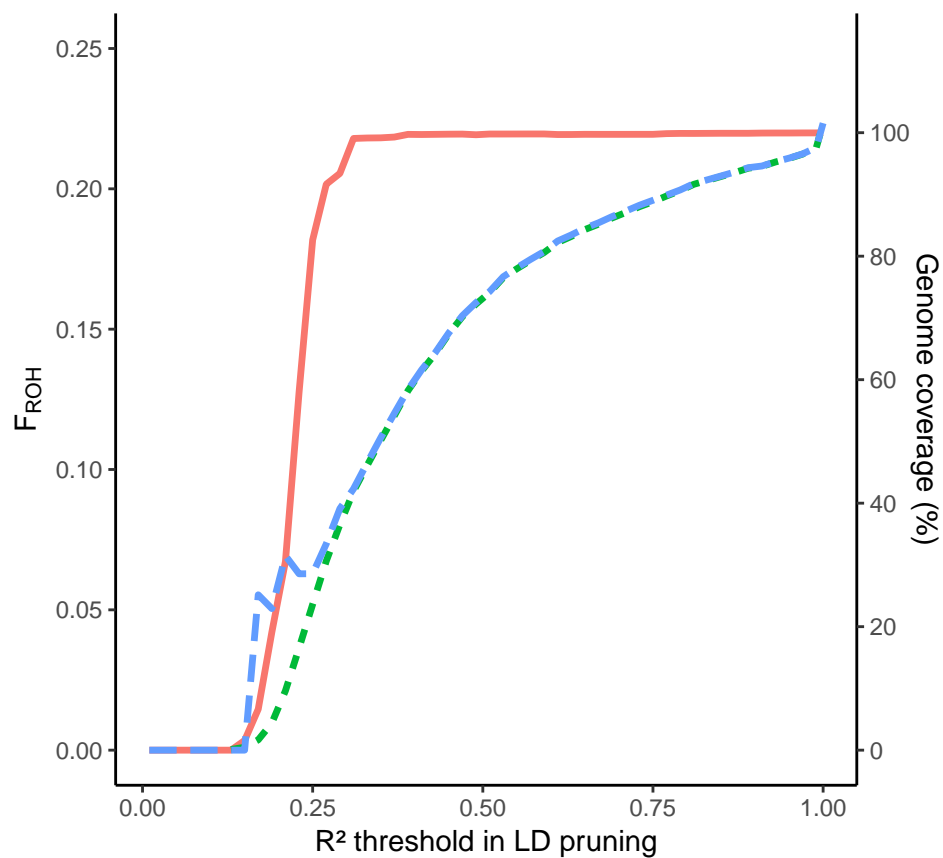

BAR

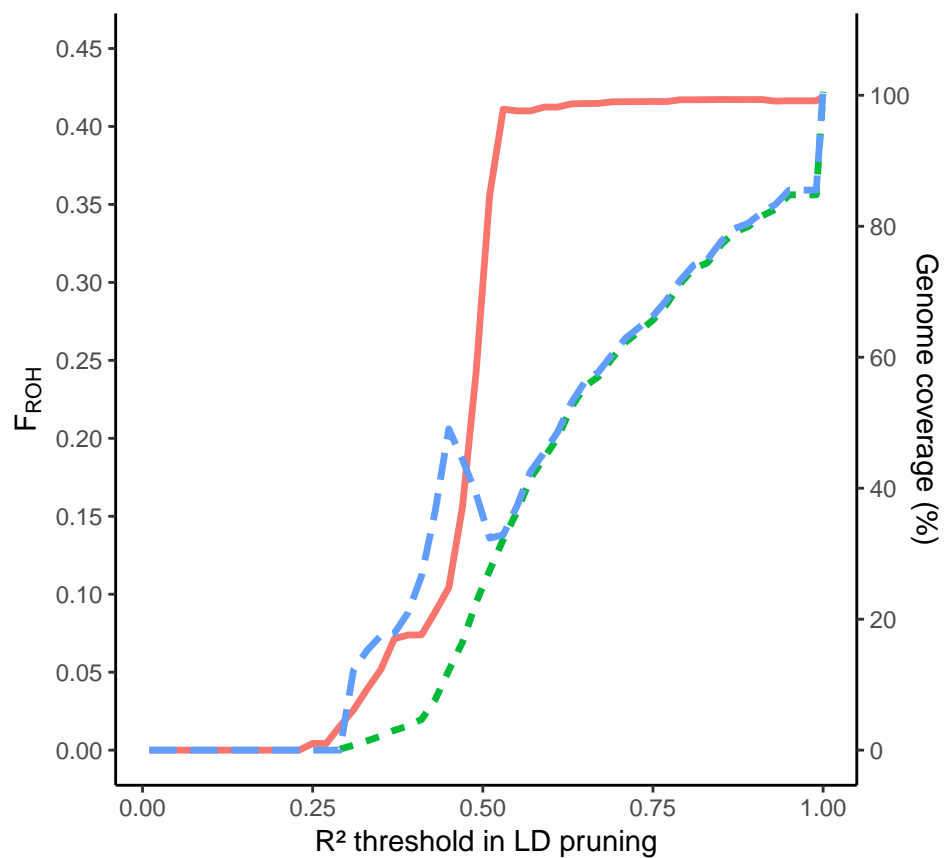

Genome coverage  $F_{ROH}$  aut  $F_{ROH}$  cov

Supplement: Supplementary file 2 — Additional file 2: Figure S1. The effect of linkage disequilibrium (LD) pruning on genome coverage and FROH estimates for ICE, SAA, LAB and BAR in PLINK. [file 12864_2020_6463_MOESM2_ESM.pdf]

**ICE**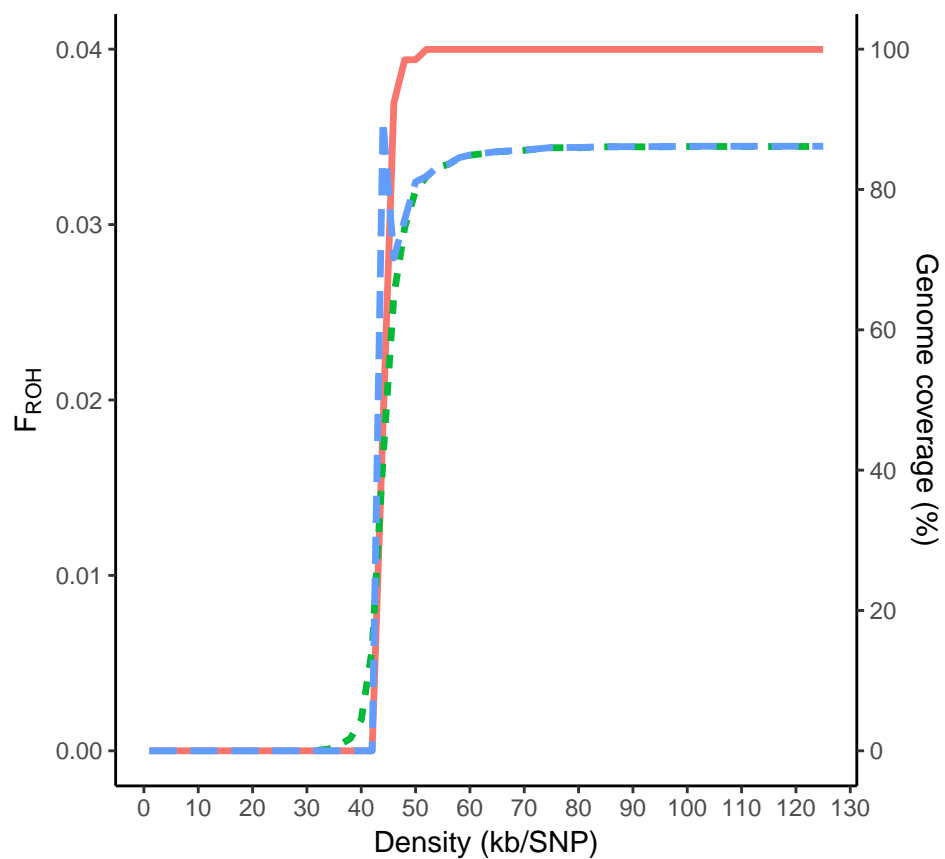**SAA**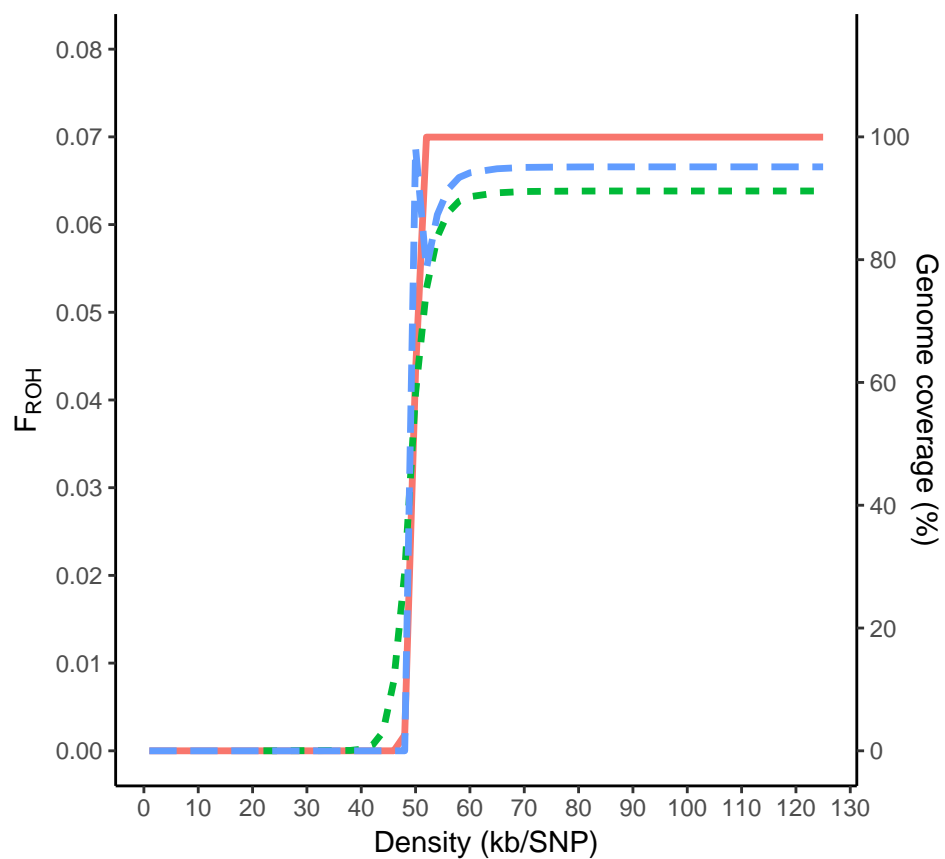**LAB**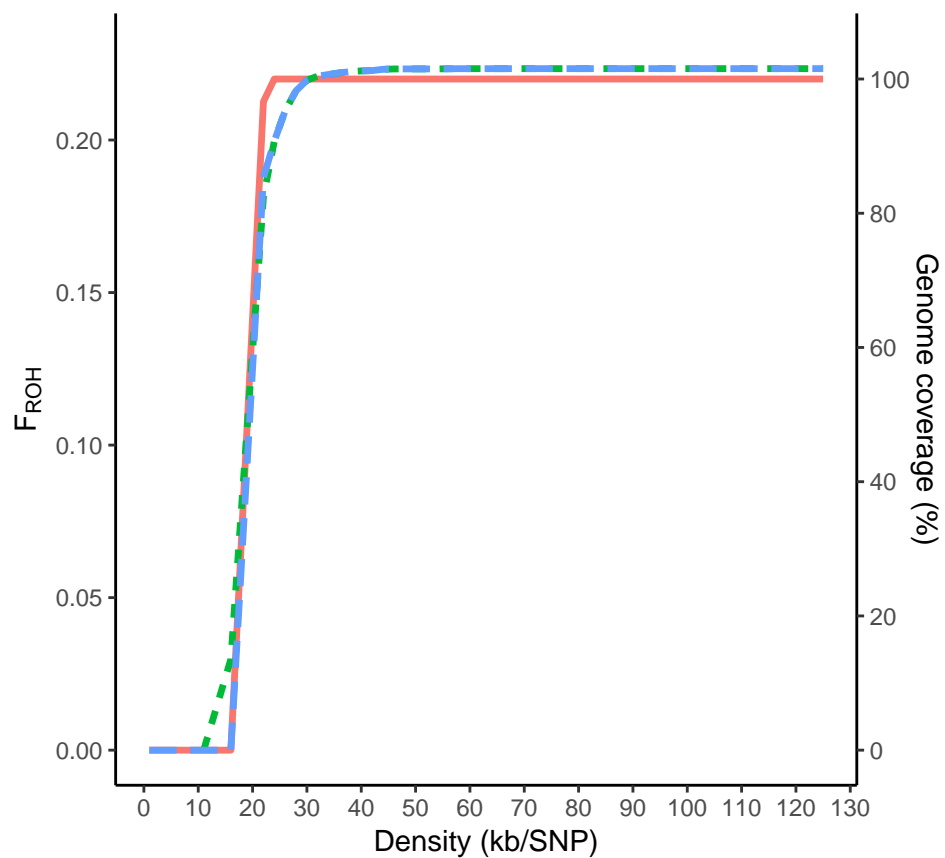**BAR**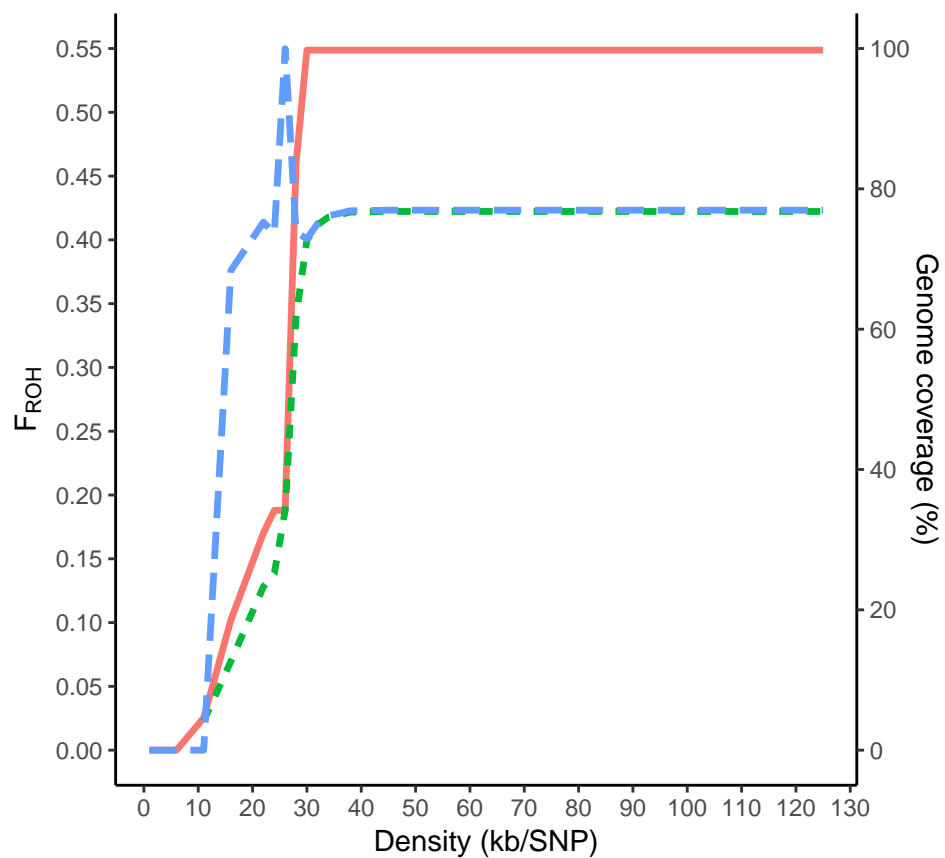

— Genome coverage — F ROH aut — F ROH cov

Supplement: Supplementary file 3 — Additional file 3: Figure S2. The effect of the density setting (in kb/SNP) on genome coverage and FROH estimates for ICE, SAA, LAB and BAR. [file 12864_2020_6463_MOESM3_ESM.pdf]

ICE

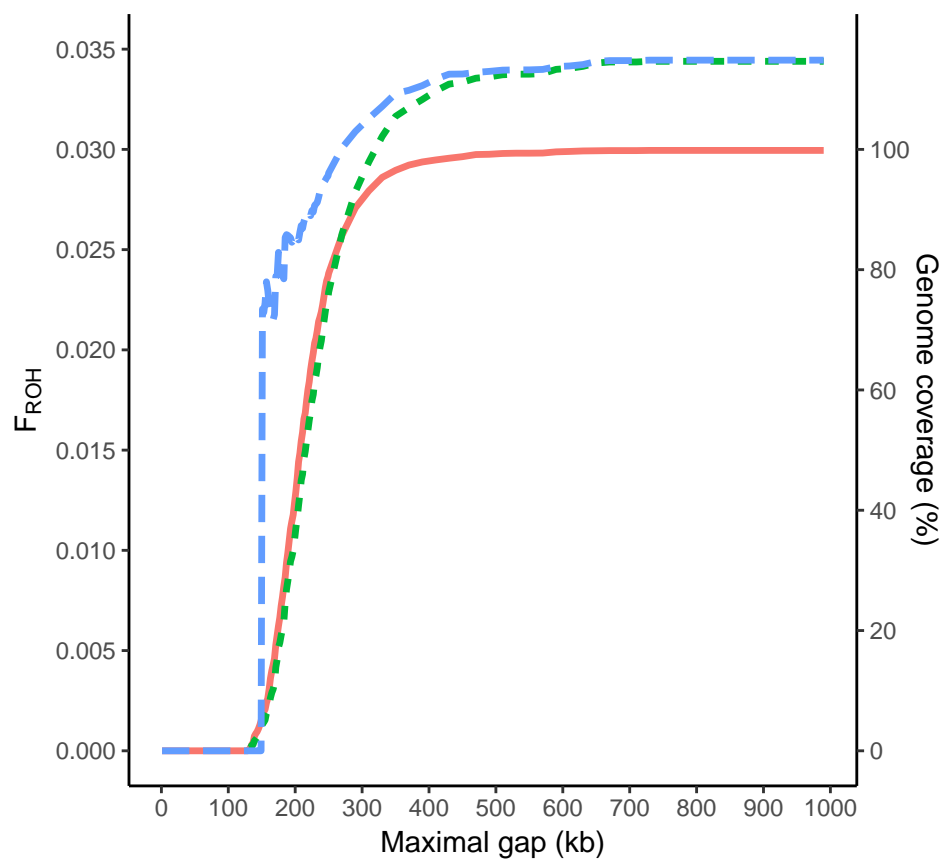

SAA

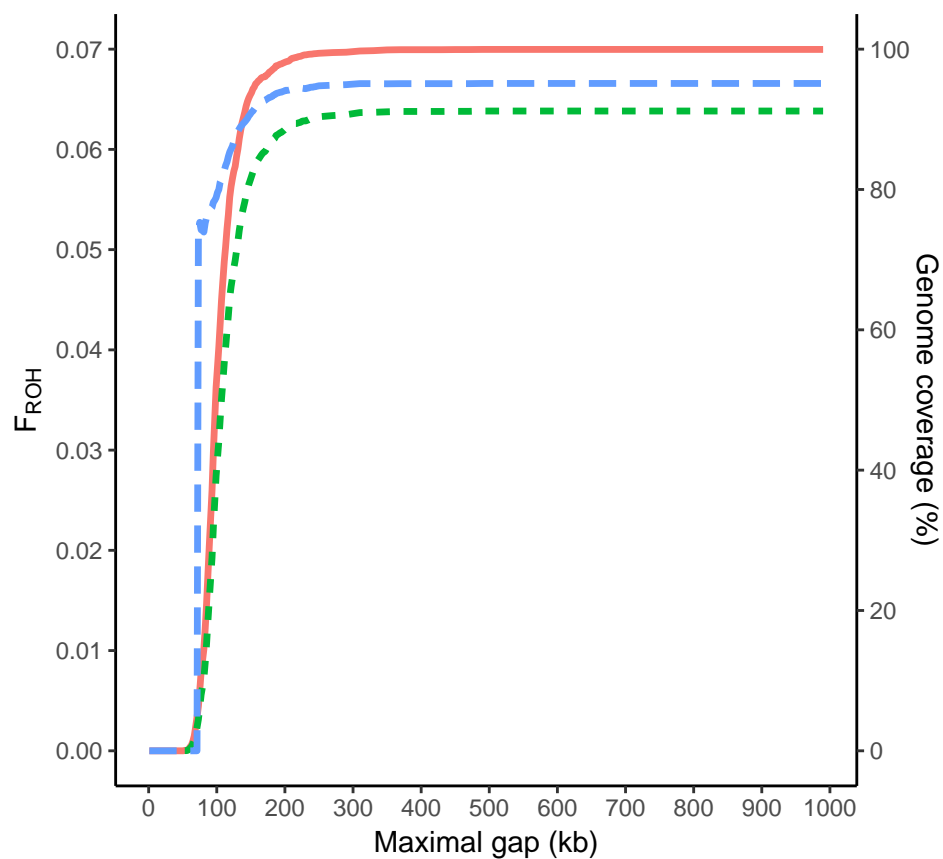

LAB

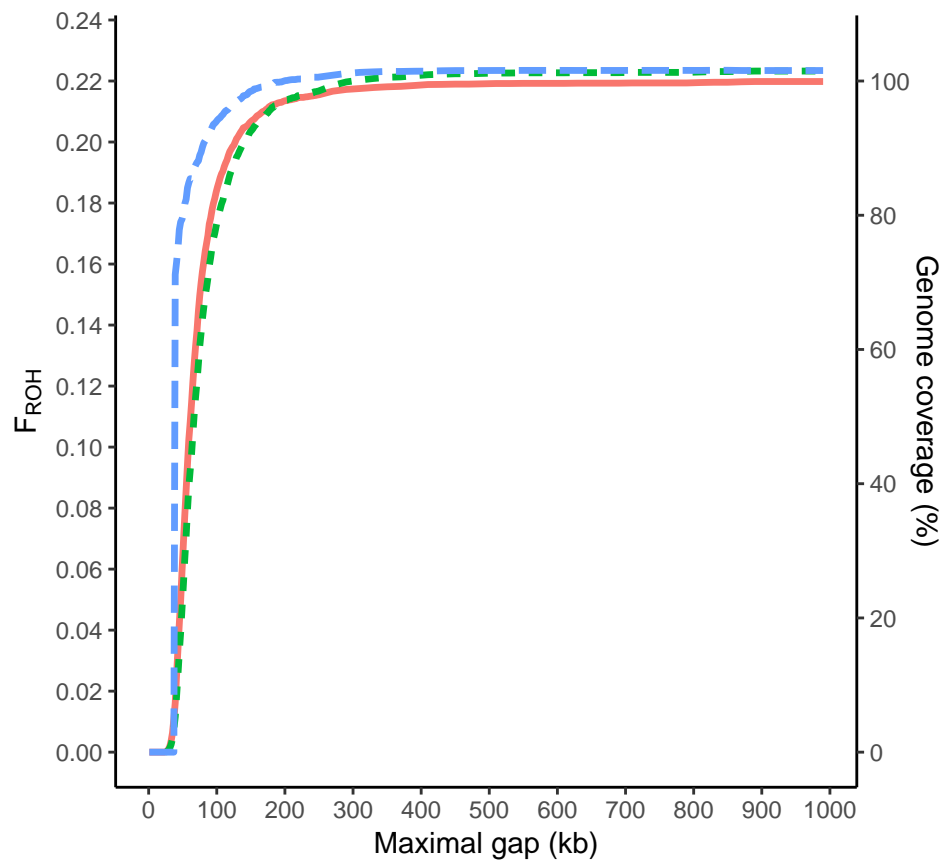

BAR

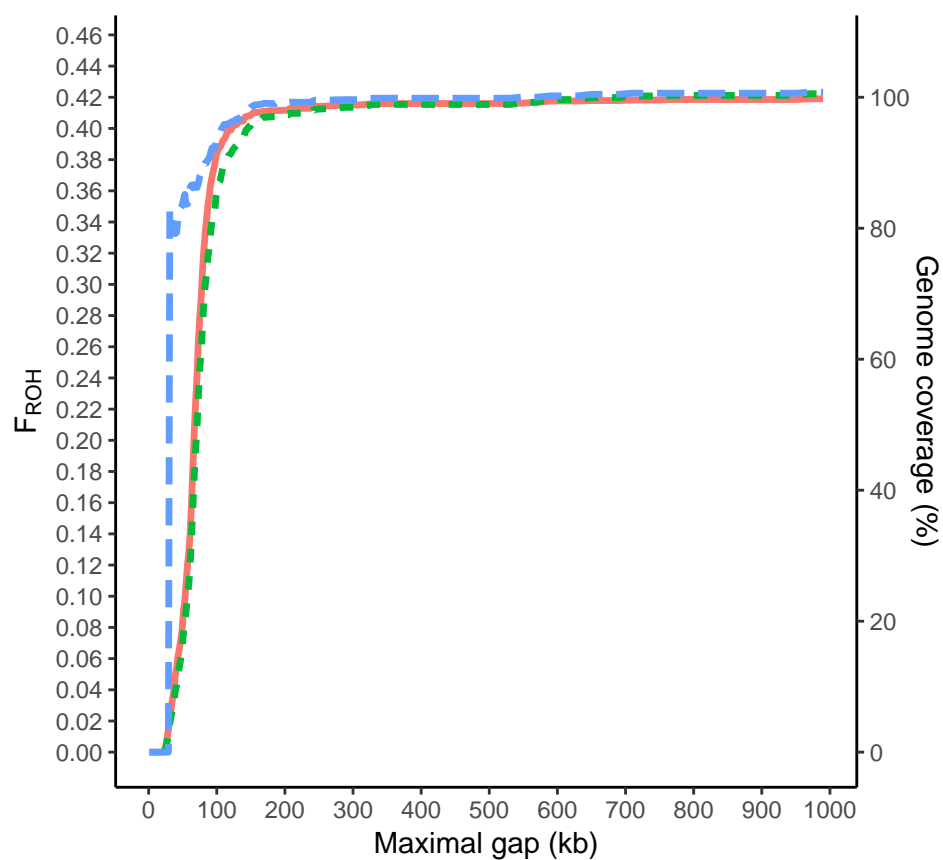

— Genome coverage —  $F_{ROH}$  aut —  $F_{ROH}$  cov

Supplement: Supplementary file 4 — Additional file 4: Figure S3. The effect of maximal gap setting (in kb) on genome coverage and FROH estimates for ICE, SAA, LAB and BAR. [file 12864_2020_6463_MOESM4_ESM.pdf]

PIT

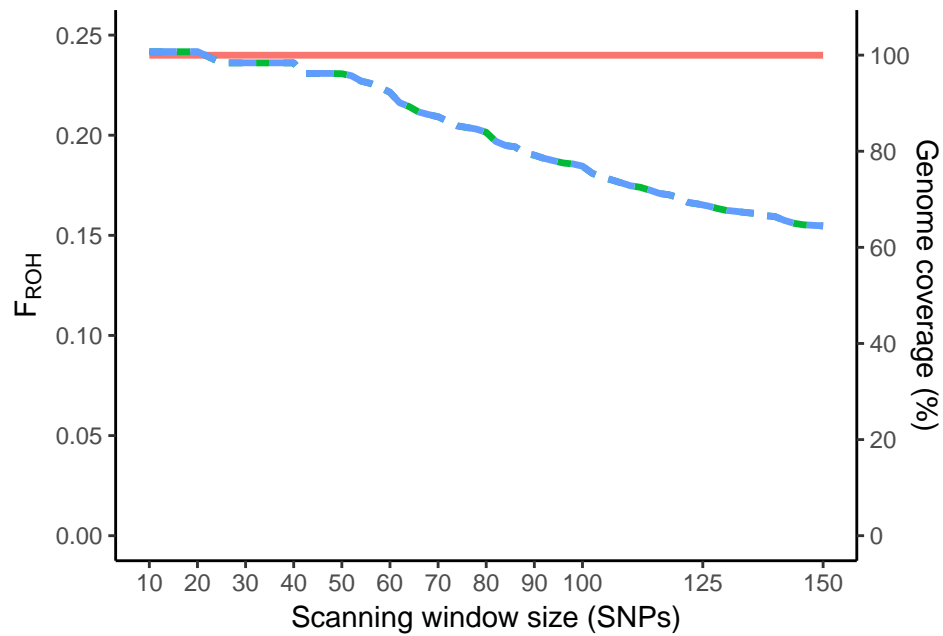

BB

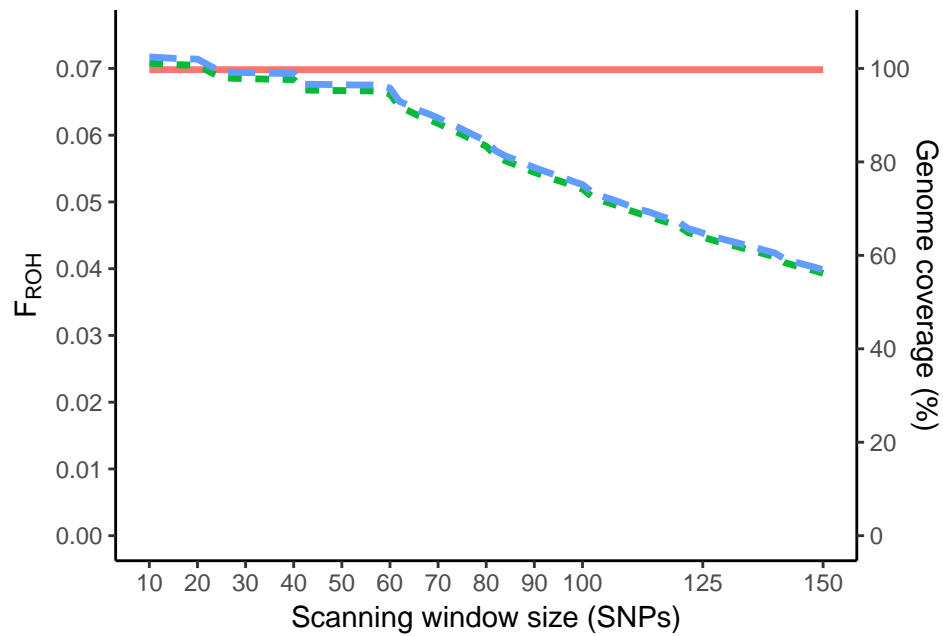

MER

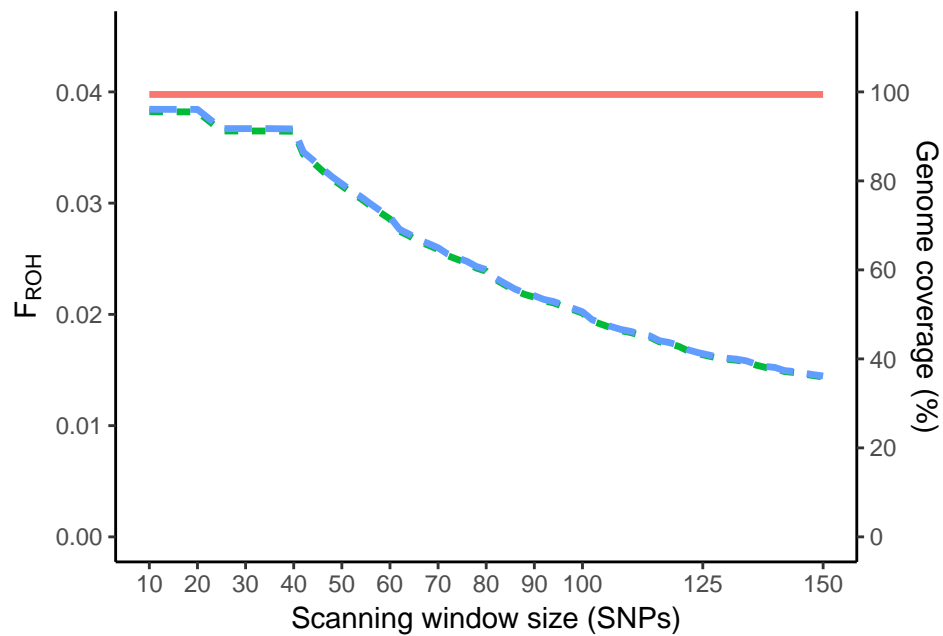

BUR

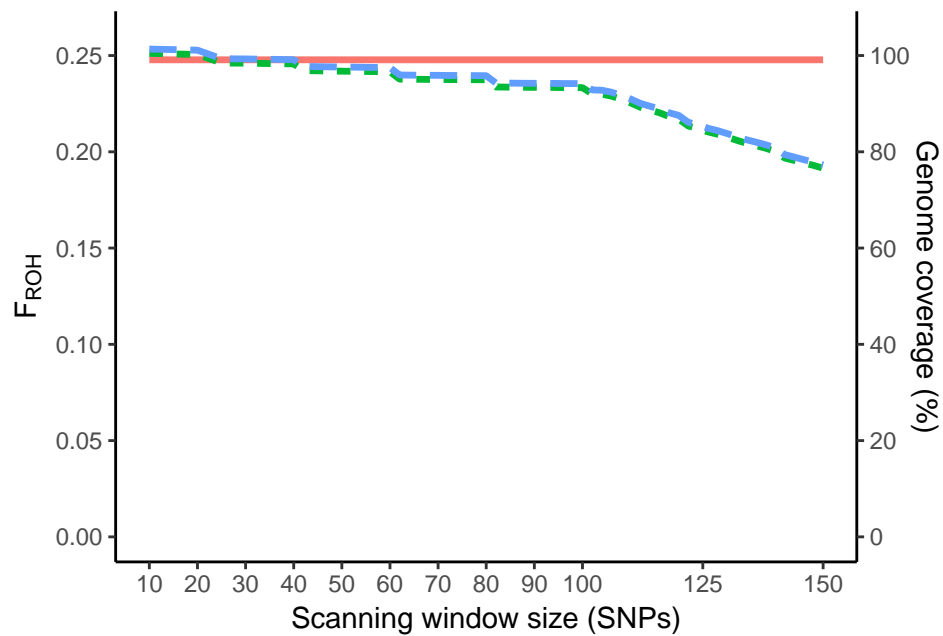

ICE

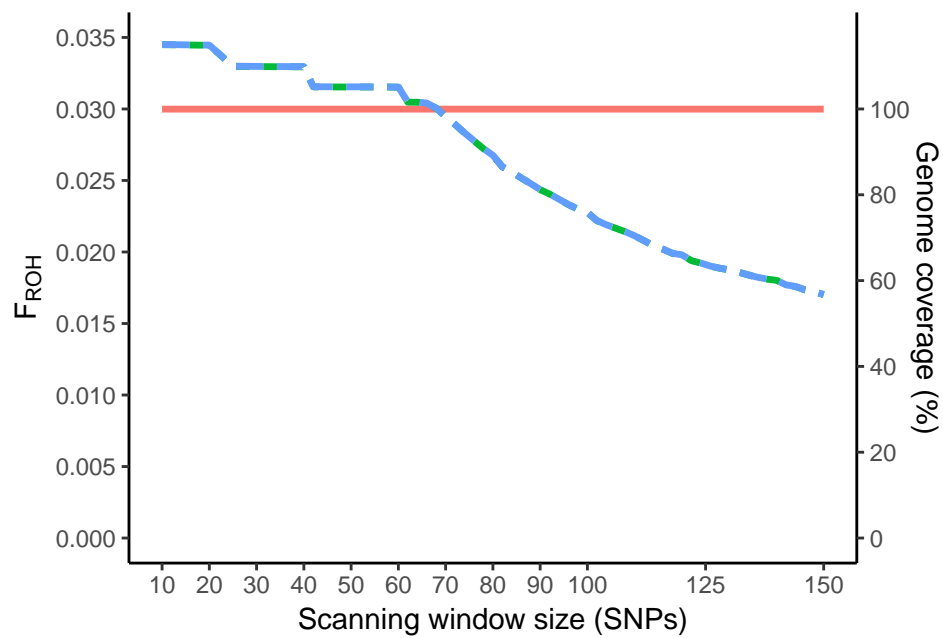

SAA

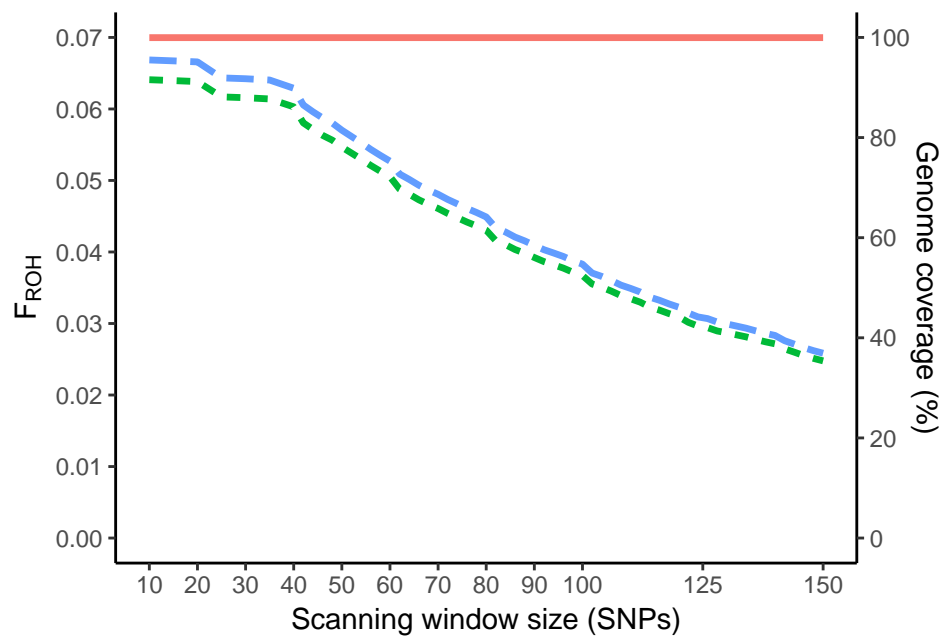

LAB

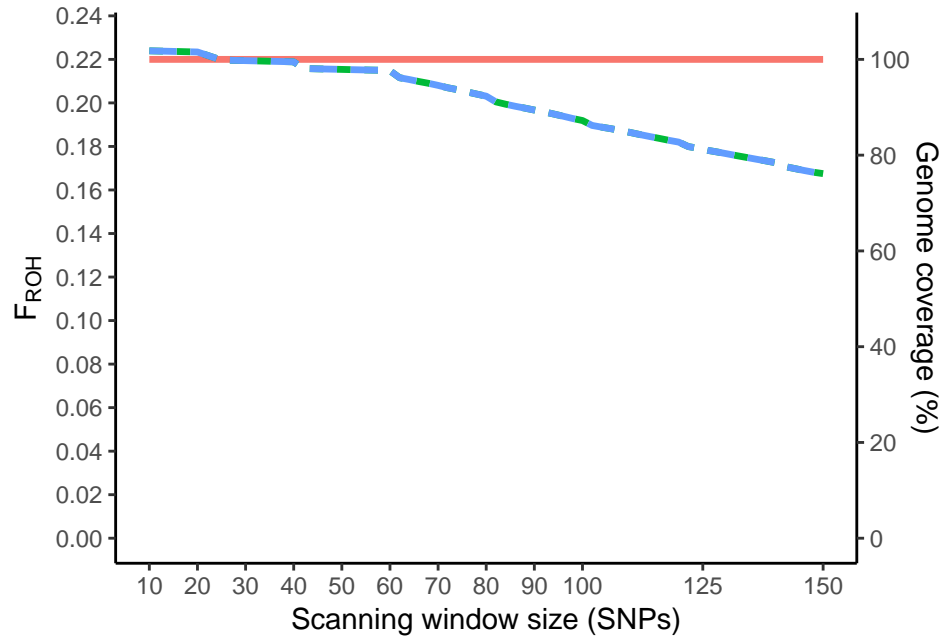

BAR

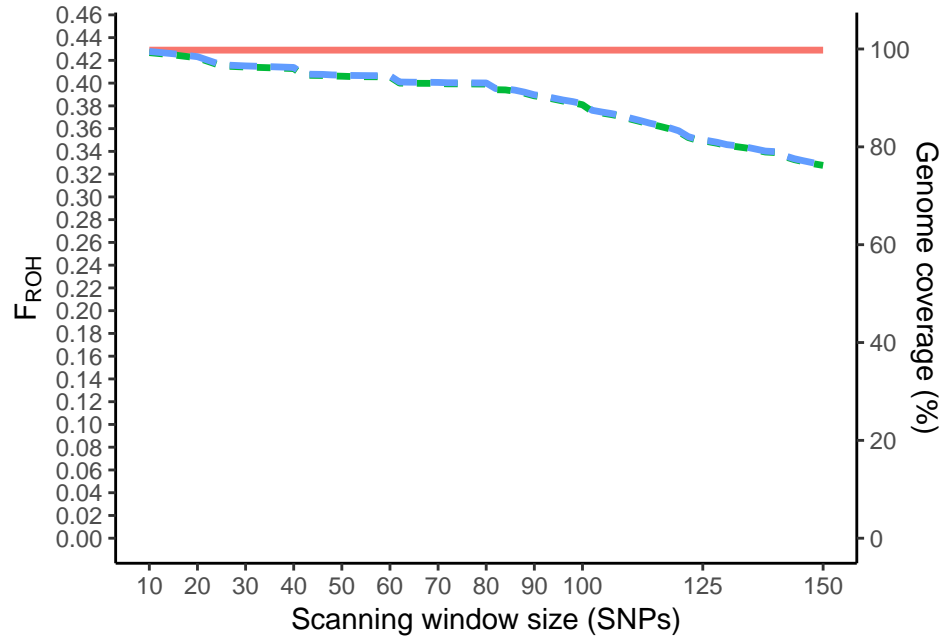

Genome coverage F ROH aut F ROH cov

Supplement: Supplementary file 5 — Additional file 5: Figure S4. The effect the scanning window size on genome coverage and FROH estimates for all evaluated populations. [file 12864_2020_6463_MOESM5_ESM.pdf]

PIT

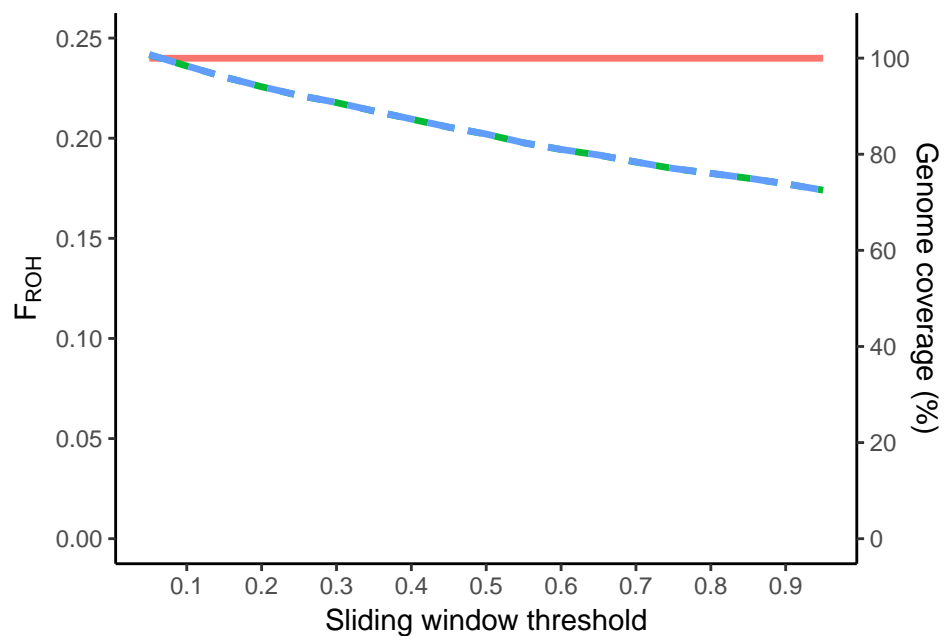

BB

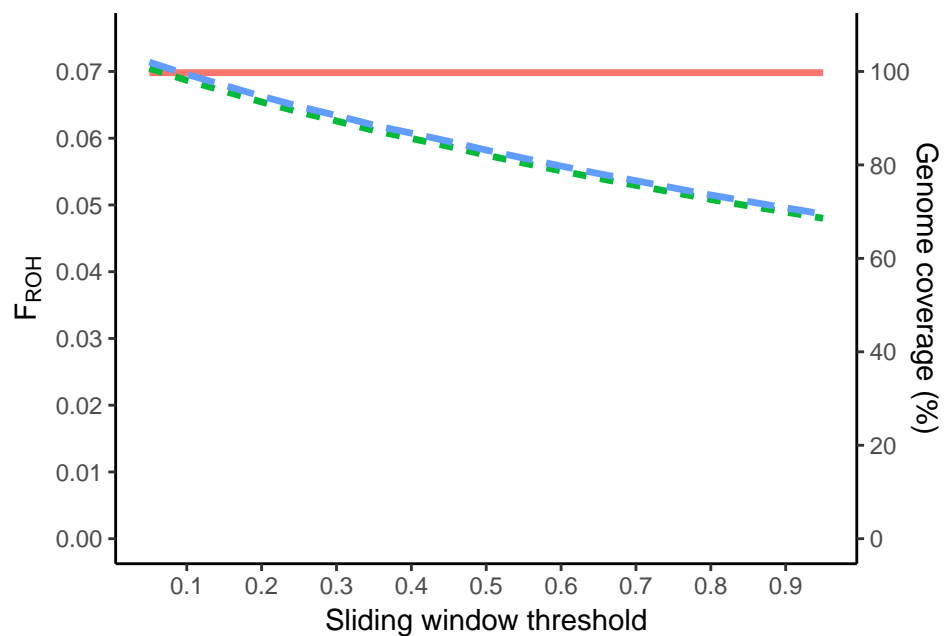

MER

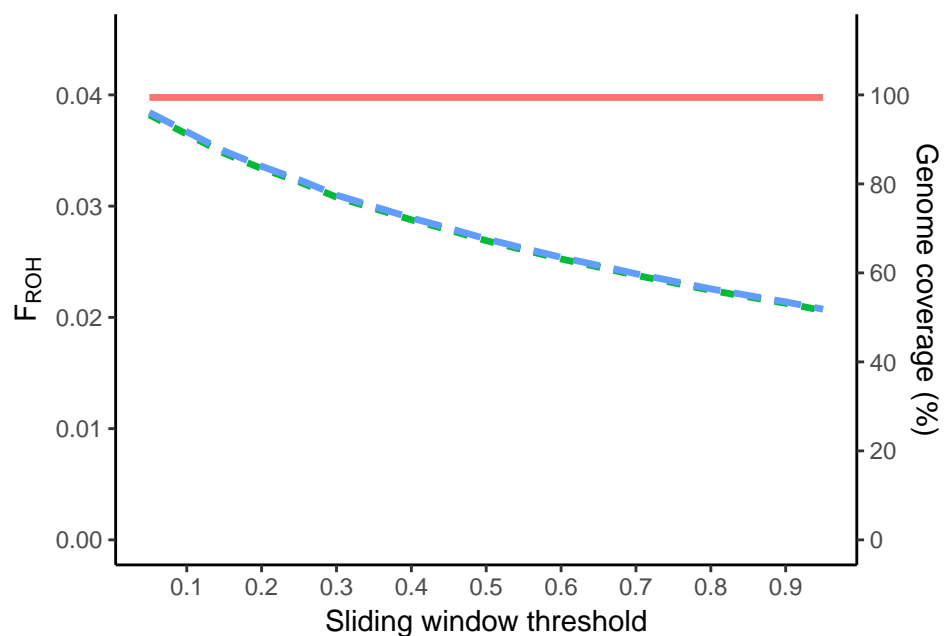

BUR

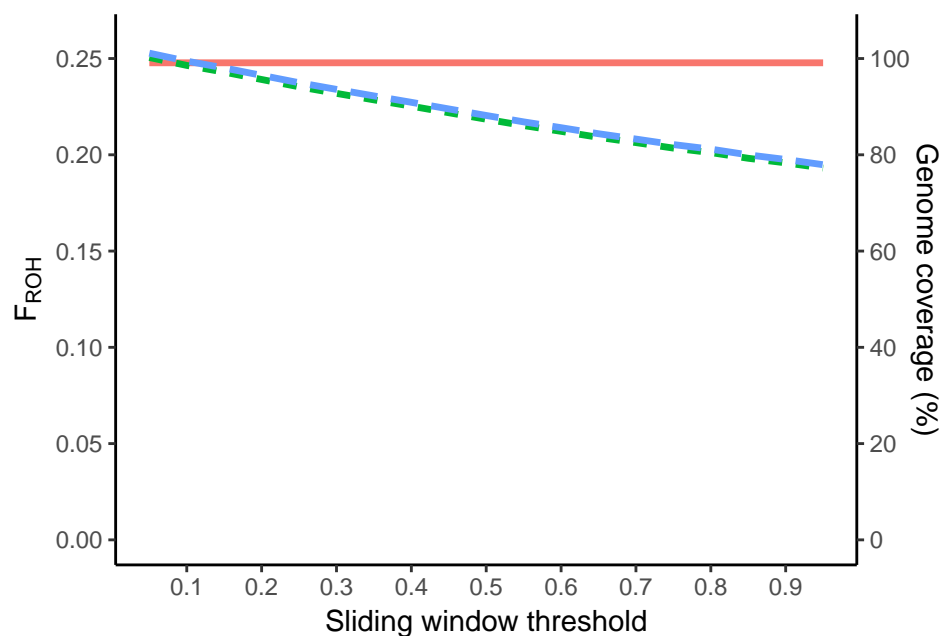

ICE

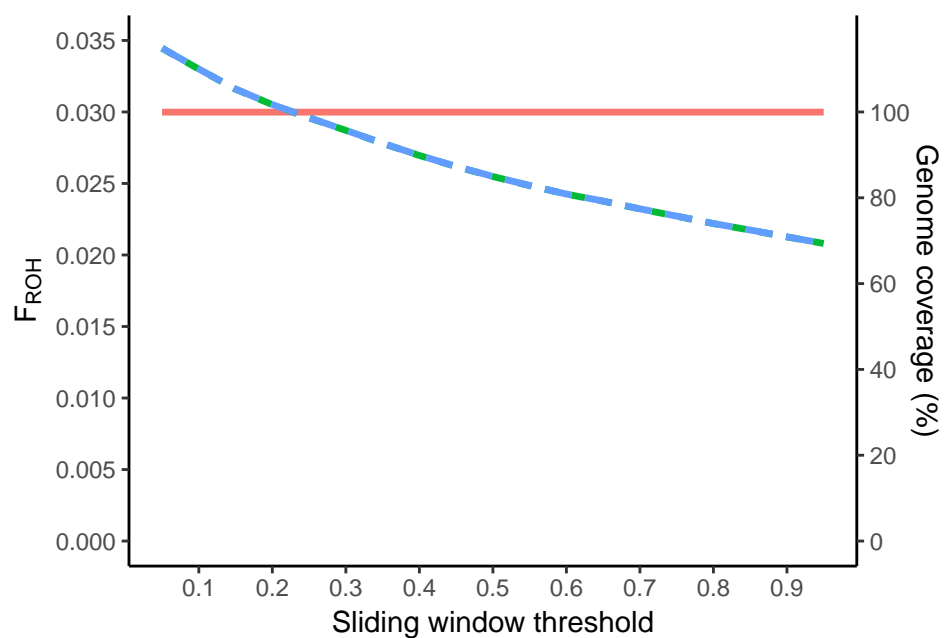

SAA

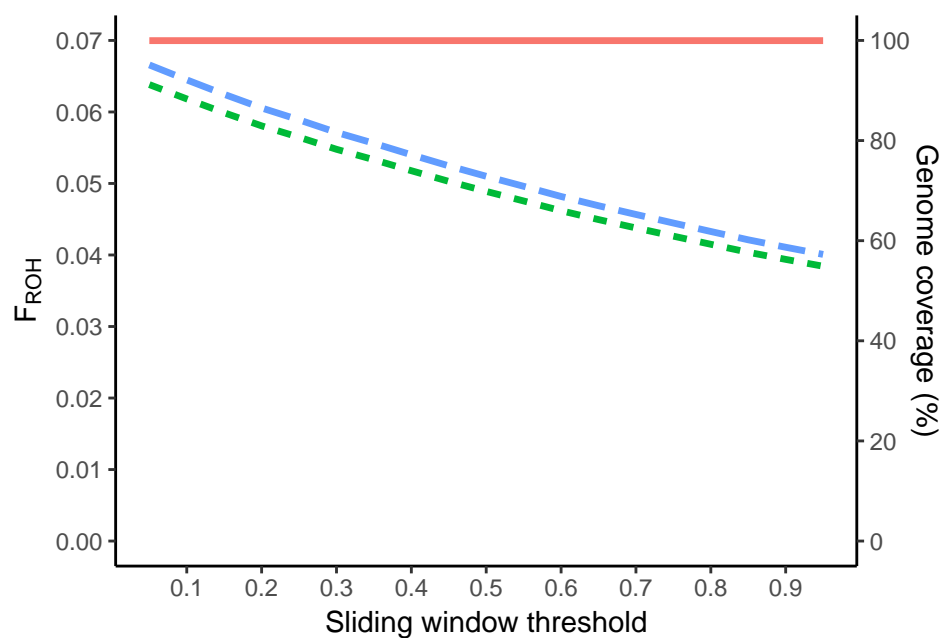

LAB

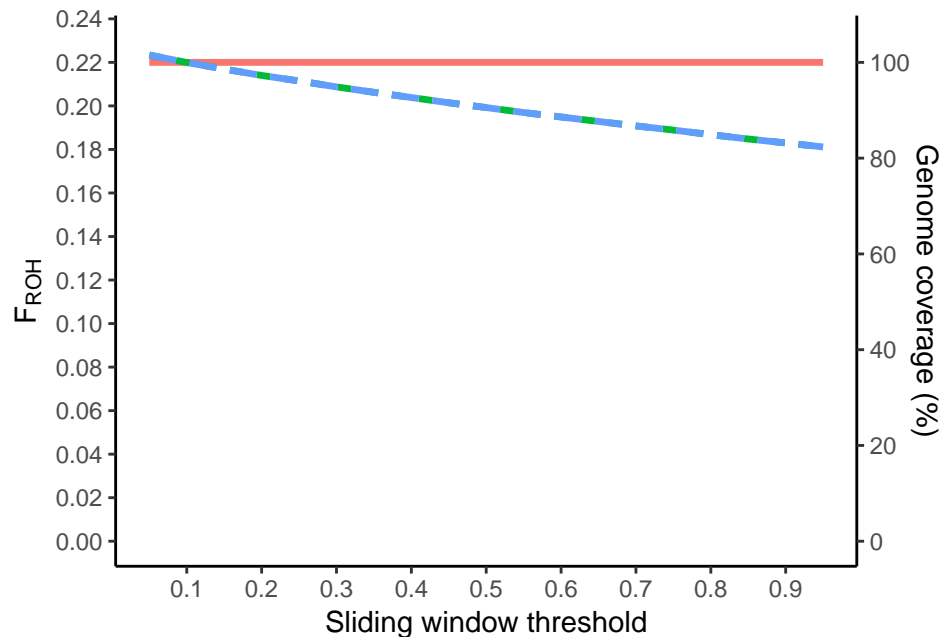

BAR

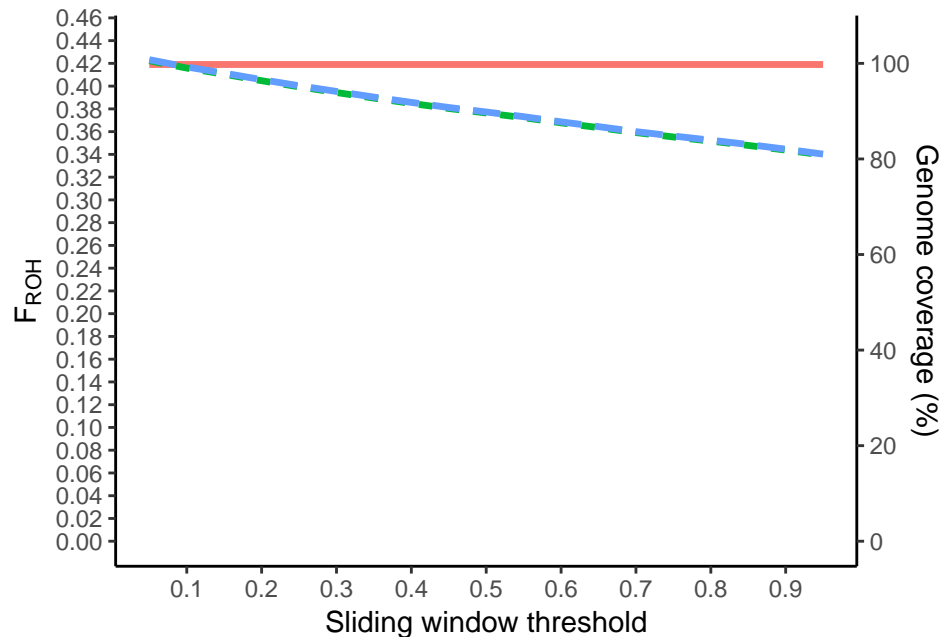

Genome coverage F ROH aut F ROH cov

Supplement: Supplementary file 6 — Additional file 6: Figure S5. The effect of the scanning window threshold on genome coverage and FROH estimates for all evaluated populations. [file 12864_2020_6463_MOESM6_ESM.pdf]

ICE

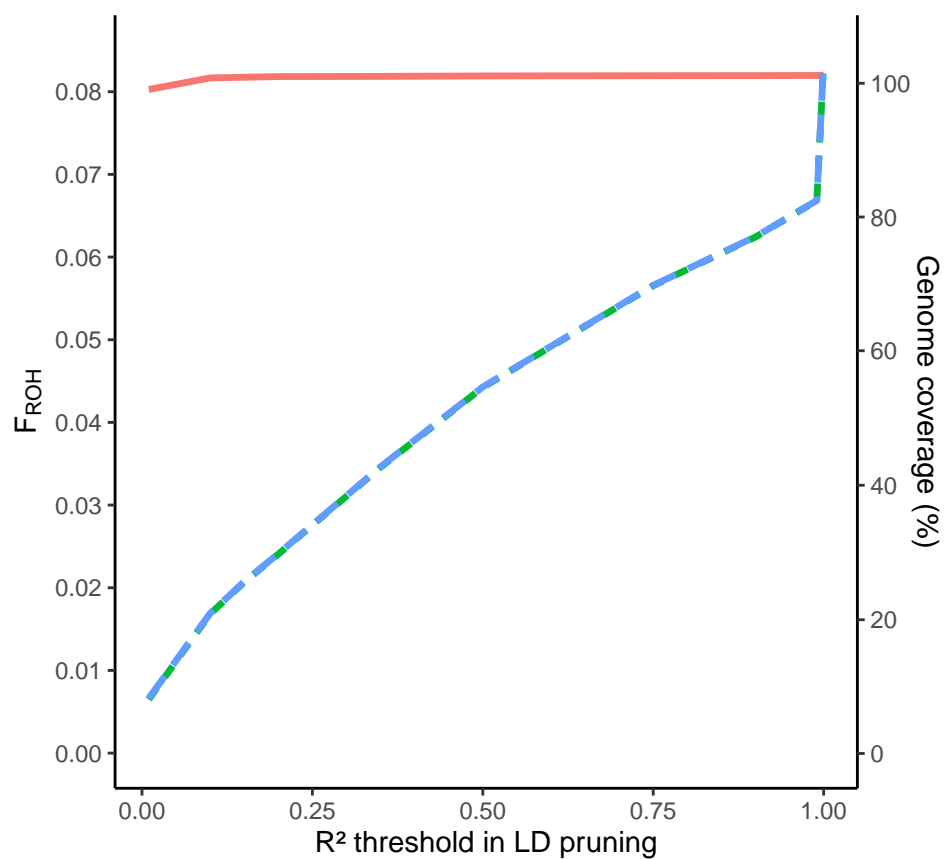

SAA

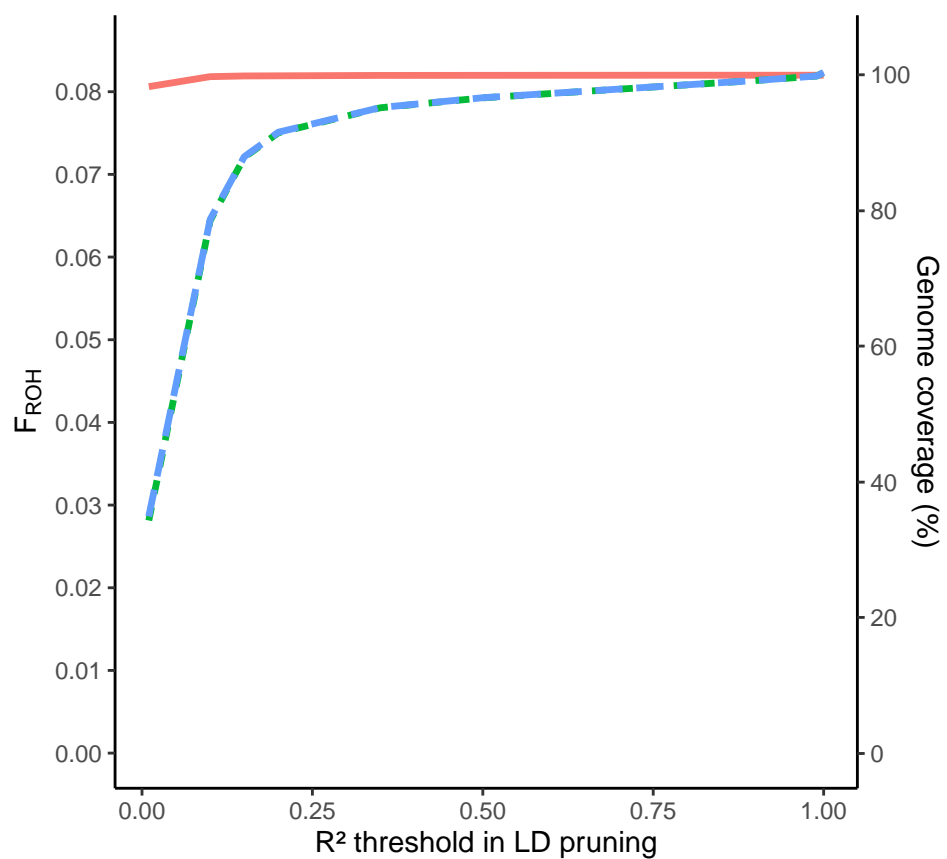

LAB

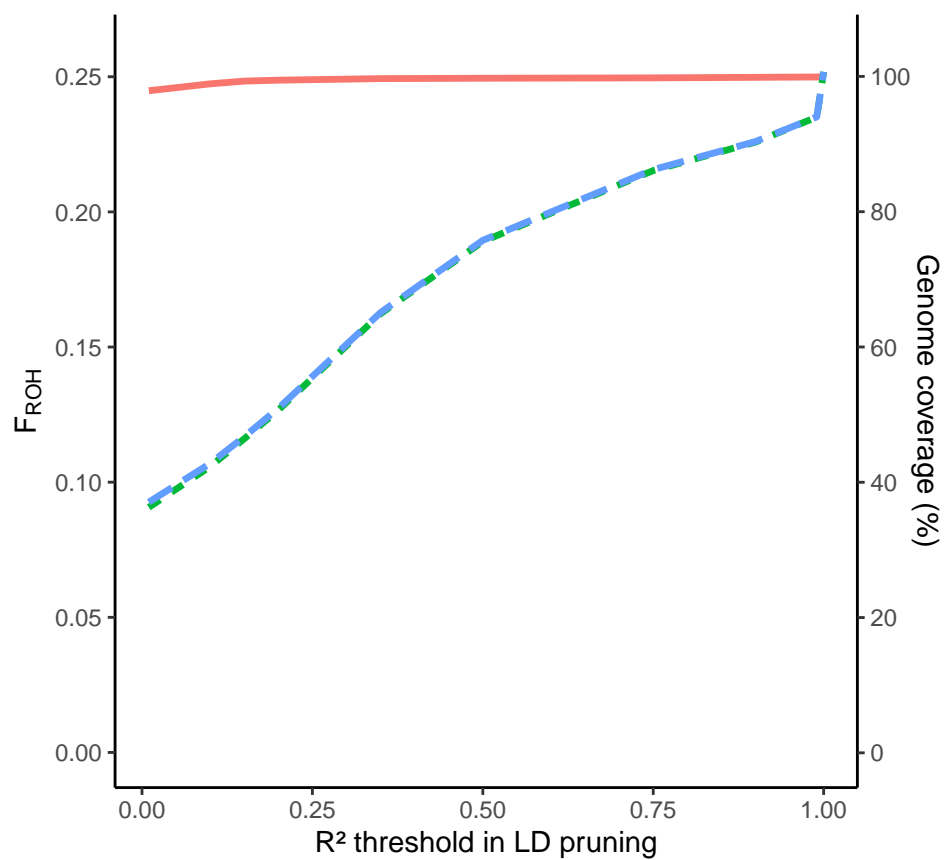

BAR

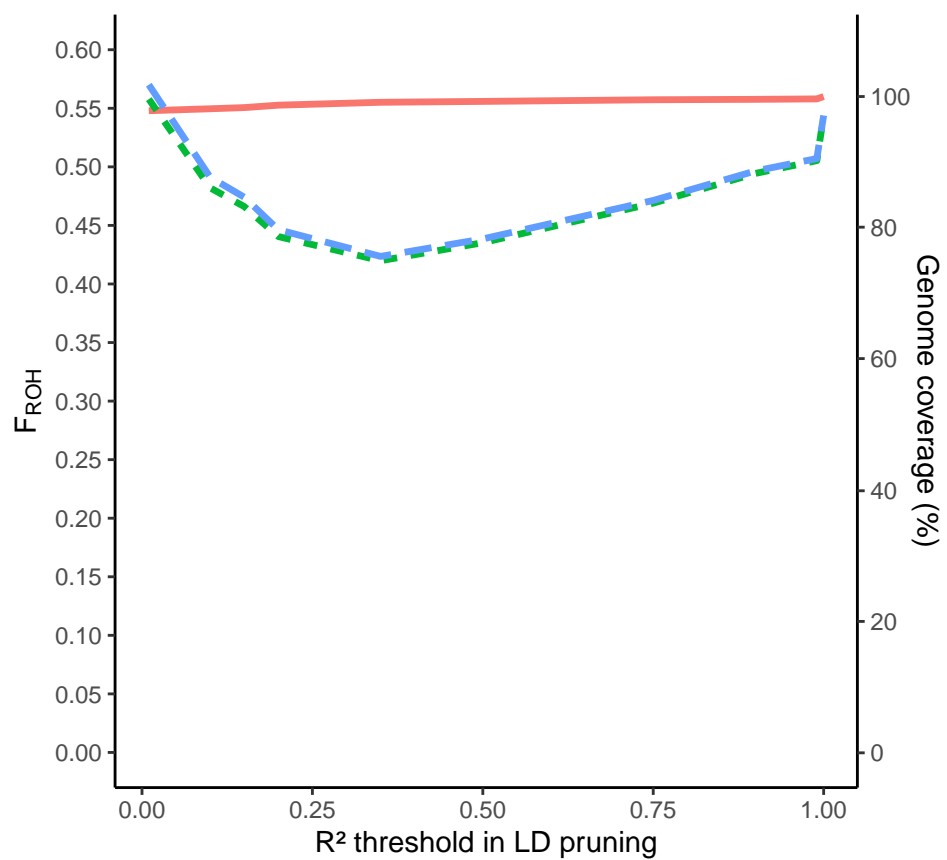

— Genome coverage — F<sub>ROH</sub> aut — F<sub>ROH</sub> cov

Supplement: Supplementary file 7 — Additional file 7: Figure S6. The effect of linkage disequilibrium (LD) pruning on genome coverage and FROH estimates for ICE, SAA, LAB and BAR in RZooRoH. For BAR, an increase in FROH was detected at very low R2 values (> 0.35), probably linked to a high degree of inbreeding and a strong decrease in number of markers in a small genome to reliably estimate HBD. [file 12864_2020_6463_MOESM7_ESM.pdf]
